# Experimental Validation and Prediction of Super-Enhancers: Advances and Challenges

**DOI:** 10.3390/cells12081191

**Published:** 2023-04-19

**Authors:** Ekaterina V. Kravchuk, German A. Ashniev, Marina G. Gladkova, Alexey V. Orlov, Anastasiia V. Vasileva, Anna V. Boldyreva, Alexandr G. Burenin, Artemiy M. Skirda, Petr I. Nikitin, Natalia N. Orlova

**Affiliations:** 1 Prokhorov General Physics Institute of the Russian Academy of Sciences, 38 Vavilov St., 119991 Moscow, Russia; 2 Faculty of Biology, Lomonosov Moscow State University, Leninskiye Gory, MSU, 1-12, 119991 Moscow, Russia; 3 Faculty of Bioengineering and Bioinformatics, Lomonosov Moscow State University, GSP-1, Leninskiye Gory, MSU, 1-73, 119234 Moscow, Russia

**Keywords:** super-enhancer, chromatin looping, therapy, regulation, experimental validation, drugs targeting chromatin interactions

## Abstract

Super-enhancers (SEs) are cis-regulatory elements of the human genome that have been widely discussed since the discovery and origin of the term. Super-enhancers have been shown to be strongly associated with the expression of genes crucial for cell differentiation, cell stability maintenance, and tumorigenesis. Our goal was to systematize research studies dedicated to the investigation of structure and functions of super-enhancers as well as to define further perspectives of the field in various applications, such as drug development and clinical use. We overviewed the fundamental studies which provided experimental data on various pathologies and their associations with particular super-enhancers. The analysis of mainstream approaches for SE search and prediction allowed us to accumulate existing data and propose directions for further algorithmic improvements of SEs’ reliability levels and efficiency. Thus, here we provide the description of the most robust algorithms such as ROSE, imPROSE, and DEEPSEN and suggest their further use for various research and development tasks. The most promising research direction, which is based on topic and number of published studies, are cancer-associated super-enhancers and prospective SE-targeted therapy strategies, most of which are discussed in this review.

## 1. Introduction

The role of transcriptional regulation mediated by super-enhancers attracted the attention of many research groups during the past decade. Since the introduction of the term, the discussion around super-enhancers has heated up, and the concept of such a cis-regulatory mechanism has not been clearly and completely defined [1,2].

In 2013, the term “super-enhancer” (SE) was suggested and proposed as a novel regulatory class represented by large clusters of enhancer elements with abnormally high transcription factor enrichment and target gene expression. The definition of SE includes a list of characteristic features that were found to correlate with the occurrence of active super-enhancers within various cell lines. The majority of in vitro studies emphasize the local enrichment of master transcription factors (MTFs), boosted epigenetic modifications (H3K4me1, H3K27ac etc.) along with the grouping of more than two enhancers (maximum distance between enhancer elements was proposed to be 12.5 kb), and high binding rate with Mediator complex subunits (especially with MedI subunit) [3,4]. These characteristics all together became the classical interpretation of the nature of SEs and formed their definitive basis. The rapid development of experimental approaches, including next generation sequencing (NGS), genome editing technologies, and the progress of computational methods, provided important insights in the structure of SEs, their impact on cell fate, and their role in the pathogenesis of various diseases. It has been shown that super-enhancers could largely determine cells’ identity during normal development and differentiation as well as in pathological states due to strong key gene upregulation.

A number of experimental studies demonstrated contradictions with the classical definition of super-enhancers [5]. There is a discussion about the characterization relevance of super-enhancers as independent objects [6]. In this review, we focus on experimental studies about super-enhancer regulation in order to develop a stable sense of its role in tumorigenesis, cell development, differentiation, and motility. In addition, we summarize the existing computational methods used for SE detection and prediction in order to consolidate actual machine learning (ML), linear, and ChIP-Seq based approaches. Thus, the experimental data and in silico methods combined represent a promising direction in the development of preventive therapy strategies.

In order to include the latest information on super-enhancers as a novel regulatory element, we defined a set of rules used for publication searching and filtering. Here is, for example, the PubMed search formula:

(Super-enhancers OR super-enhancer) AND (chromatin structure OR TAD OR TADs OR chromosome conformation capture OR 3C OR 4C OR 5C OR Hi-C OR NG Capture-C OR distant interactions OR looping OR 3D OR 3D structure) [Publication Date]

Similar queries were formulated in order to access papers from the following research databases: Scopus, Web of Science, EMBASE, GHL, VHL, Cochrane, Google Scholar, mRCTs, POPLINE, and SIGLE.

Found papers were further classified as relevant and used for the qualitative summary if any of the following rules were applicable: (i) article contains specific information associated with super-enhancers; (ii) is an in silico research article with various SE-search algorithms applications (pipeline must be available for robustness evaluation and result reproducibility); (iii) is an in vivo/in vitro research article followed by a well-described methods section. In addition, we formulated the exclusion criteria for the set of retrieved papers: (i) duplicated work; (ii) inaccessible article; (iii) paper published earlier than 2013 (this criterion was necessary to bring up as soon as the term itself appeared only by this year); (iv) articles contrary to the inclusion rules.

## 2. Constitutive Super-Enhancers in Different Cell Types

### 2.1. Defining Constitutive Super-Enhancers: Current View

It is remarkable that the DNA molecule serves as efficient storage of genetic information for multiple cells, and that gene expression regulation results in the unique phenotype of each cell. Turning genes “on” and “off” depends on certain proteins called transcription factors (TFs) attaching to some specific short sequences of DNA (500–1500 bp long) known as enhancers. They are found in both prokaryotic and eukaryotic organisms and, though acting in cis, can be located up to 1 million base pairs away from the target sequence. The formation of SE complexes through numerous TFs binding boosts the activity of the genes nearby. A multicenter study initiative from 2011, the Encyclopedia of DNA Elements (ENCODE) [7], reported the identification of approximately one million enhancer switches in the human genome.

**Figure 1:**
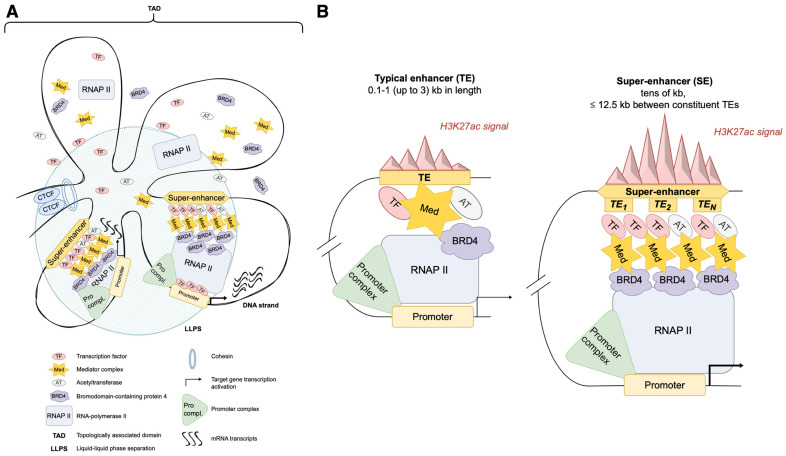
Schematic representation of the SE concept compared to the TE. (**A**). LLPS model (blue circle) inside a TAD with two differentially active SEs, which are surrounded by the elements of transcription activation complex and RNAP II. (**B**). The TE structure, being less lengthy compared to its SE analogues and comprising one distinct TE region, shows moderate levels of H3K27ac signaling.

The term “super-enhancer” was introduced by Chen and colleagues in 2004 to describe a baculoviral DNA locus, *hr3*, that could induce the transcription of *ie1* promoters up to a thousand-fold change, implying the first differentiation criterion for a SE: higher transcriptional effects [8]. Three first trendsetting studies by Young et al., published later in the 2010s, coined the term “super-enhancer” to describe extended genomic domains that play an important role in cell identity [3,5] and disease control [9] and act differently from the single enhancer or typical enhancer (TE) (Figure 1). Based on his team’s findings, Young speculated that some of these enhancers do not function alone but rather come together in large groups.

The main concept of super-enhancers’ action mechanism is very similar to one that was previously described for normal enhancers. Transcription factors bind to specific motifs of super-enhancers and facilitate SE interaction (looping model) with subunits of transcriptional complexes (RNA-pol II, Med. TFIIF, etc.) in the promoter regions of regulated genes. Therefore, they activate target gene expression through the recruitment of numerous transcription machinery units [10]. The nuances of this seemingly simple mechanism remain unclear, but there is a proposed phase separation model that could shed light on the studied SEs’ functions [11]. The model includes two parameters: the number of molecules and their “valence” in the volume of the super-enhancer. It is suggested that the transcription activity of target genes non-linearly depend on the relative amount of interaction between transcription factors within the SE volume. If the interaction value between TFs is close to maximum, then the phase separation occurs, and the probability of gene transcription grows. Thus, the balance between the concentration of TFs and valence values are crucial in terms of effective SE insulation and further target gene transcription maximization. 

Nevertheless, there are many other factors which potentially affect SE function and change the transcription probability and rate of dependent genes. For example, it is known that massive eRNA transcription from super-enhancers plays an important role during the cis-regulation of target genes [12]. At the same time, these eRNAs may modulate the expression of distant genes that are not initially associated with studied super-enhancers. Such proposed mechanisms should be carefully researched in order to gain a deeper understanding of super-enhancers’ mechanistic nature.

It was investigated that the epigenetic regulator BRD4 and the multifunctional cMyc proteins play an important role in mice with leukemia. The scientists reported that blocking Brd4 decreases Myc and prevents cancer cell proliferation, and that Brd4 binds to a 40000-bp stretch near the *MYC* gene. Further, the researchers showed that not only cancer cells but also healthy stem cells (where much of the enhancer-associated Mediator complex occupies exceptionally large enhancer domains associated with key embryonic stem cell (ESC) biology genes), muscle cells [3], and other cell types owe their unique identities to these SE gene-regulating structures. They were able to demonstrate that SEs span tens of kilobases (kb) of DNA sequences compared to TEs and are densely occupied by master transcription factors (TFs) and mediators using ChIP-seq data from multiple tissue types that were available from the Roadmap Epigenome and ENCODE projects [7,13]. In the aforementioned study, led by Jakob Loven of the Whitehead Institute, the team found that a super-enhancer is formed near the *MYC* gene and that its structure catalyzes the high production of MYC protein. They also found that super-enhancers are even more susceptible to disruption than key chromatin-remodeling proteins such as BRD4, which would give us more therapeutic potential, as BRD4 has well-defined binding pockets that can be targeted. This would also grant the possibility to focus on the master enhancers—cell-specific SEs—instead of studying dozens of them and manipulating gene expression in cells with fewer side effects.

These very first publications related to SEs caused quite a heated discussion, as did the publications of the ENCODE consortium, which succumbed to criticism due to sweeping statements proclaiming 80% of the human genome to be functional [14,15,16,17]. Young’s definition of SEs in relation to developmentally important genetic elements extend far beyond its early usage, which was based on their performance in in vitro expression assays and has since become more or less standard. Initially, super-enhancers were reported as sequences involved in the regulation of transcription, which may be more than the synergistic sum of their parts. There are many examples in which they occurred in groups before important genes and were bound by mediator protein complexes. However, it was also assumed that they have additional functional properties.

As [6] shows, no single logic exists in the field of SE definitions. The original approaches to finding/determining super-enhancer sequences were partly determined by mediator binding levels as measured with chromatin immunoprecipitation and sequencing (ChIP-seq); the topmost part of the resulting distribution would contain super-enhancers. Whyte’s definition of mouse ESC SEs comprised the following steps:

1. Enhancers were considered to be the sites bound by all three master regulators, Oct4, Sox2, and Nanog, according to ChIP-seq;
2. Enhancers within 12.5 kb of each other were stitched together to form a single entity;
3. The stitched SE entities and the remaining TEs were then ranked by the total background-normalized level of Med1 signaling within the locus.

However, this definition has been criticized because it was not “functional” in essence and did not reflect the unique properties of so-called super-enhancers. Moreover, the clustering procedure in step 2 is algorithmic, implying no specific selection or filtering. Additionally, multiple publications that have defined SEs (and sometimes even within a single paper) deviate from Whyte’s group’s definition, including various characterization marks at different algorithm steps. For example, in a study by Hnisz et al. [5], H3K27ac23 was used to identify enhancer regions, whereas another group used Med1 [9]. Thus, the only defining feature of super-enhancers is an exceptionally high degree of enrichment of transcriptional activators or chromatin marks as determined by ChIP-seq, which is assessed in step 3. The major differences between normal enhancers and super-enhancers are inevitably dominated by features associated with the ChIP-seq enrichment used to define them.

It should be noted, however, that H3K27ac may be functionally dispensable for transcription, even for genes associated with super-enhancers, as it was addressed in mouse ESCs [18]. The other study led by the Huggs group [19] had a look at one of the most consistent SEs associated with the α-globin gene [20]; the authors reported comprehensive functional dissection of the SE in erythroid cells (individually and in combinations) with no significant features of synergistic or higher-order effects.

It should be mentioned that the related term “stretch enhancer” has been described in parallel [21,22]. Stretch enhancers are defined by ChIP-seq on the basis of several chromatin marks being presented over a span of 3 kb genomic loci. In some papers, the term is used as a synonym for SE and shares some of the same characteristics [23]. At the same time, one might face a paper in which SEs and stretch enhancers are distinguished. Accordingly, SEs have been shown to be transcriptionally more active and cell type-specific than stretch enhancers. A comprehensive analysis comprising histone modification and chromatin accessibility profiling as well as cell type-specific gene expression evaluation has revealed that at the genome scale, stretch enhancers are more abundant and are further away from TSS than super-enhancers, whereas super-enhancers are more evolutionarily conserved [24].

The analytical work comparing two previous experimental datasets of Hay and Shin’s groups proved that all the knockout data for *α-globin* and *Wap* SEs can be explained with a simple model [25], suggesting little evidence to support the existence of a more complex genetic element such as a SE, or at least no urgent need to develop sophisticated mechanistic models.

The controversy of the usage of the term “super-enhancer” has had a negative impact on drug discovery. Some researchers believe that super-enhancers play a particularly important role in stem cell differentiation and oncogenic pathways [26], two dominant areas of medical interest. In addition, there have been proposals to either try to attack these transcription pathways directly (which still seems to be a difficult task) or to use the characteristics of SEs for possible new drug target prioritization. Another limiting factor for drug research is data consolidation and processing. In order to ease the perspective of SE-targeted drug development, several research groups provide SE databases of various sizes and completeness. Current SE databases available include dbSUPER [27], SEA version 3.0 [28], SEdb [29], and human SEs repository (https://sunlightwang.github.io/Super-Enhancers/ (accessed on 6 March 2023)), each with comparative and exploratory analyses of publicly available ChIP-seq data. The most volumetric database, SEdb, contains up to 331,000 SEs derived from 541 human cell lines/tissues [30], and 52 SEs of the newest SEA v. 3.0 have been experimentally confirmed.

Still, there have not been enough studies disclosing the evolutionary stability of super-enhancers as separate biological units (which would be interesting to do on a variety of related model lines, such as in healthy vs. tumor cells). In the current concept, the definition of a super-enhancer could be formulated as follows: super-enhancers in a broad range of human cell types are (putative) extensive clusters of enhancers with aberrantly high levels of transcription factor binding, which are historically associated with higher expression rates of cell type specification genes (including cell identity and pro-oncogenic genes). In order to provide the most comprehensive definition of SEs while comparing them to typical enhancers and with reference to published data, we provide a summary table with the main feature characteristics for both regulatory elements (Table 1).

The algorithms used to identify typical enhancers and super-enhancers differ in their complexity and the number of genomic features they analyze. While both methods rely on ChIP-seq data for histone modifications, the identification of super-enhancers requires additional data and more complex algorithms along with the tuning of the latter. There are various proposed metrics that could be possibly used for SE identification along with machine learning methods with similar precision and validity. One of these involves a super-enhancer’s frequency across various cell lines. There is emerging evidence for a class of SEs that seem to be universally active; they display a strong association with fast recovering chromatin loops after sequential cohesin removal and restoration [31] and reveal a constitutive occurrence pattern (or “constitutively active”, or “common”) and extra high degree of universality across different cell and tissue types.

### 2.2. Techniques Used and Proposed for SE Discovery and Research

To study the biology of super-enhancers, the mechanisms of their functioning, their role in a particular cell type, and the effect of various agents in particular inhibitors, a set of both computational and experimental methods is needed. In order to study super-enhancers, they are often used in tandem. Bioinformatics methods are used to process experimental data and search for potential objects, and experimental data itself could be used to refine in silico predictions. Here, we review bioinformatic methods for searching for super-enhancers, focusing on machine learning methods and experimental techniques that have been actively used in recent years.

#### 2.2.1. Computational Methods for Searching and Predicting Super-Enhancers

To solve the problem of searching for super-enhancers, classical methods and machine learning (ML) approaches, including neural networks (NN), were used. Most of the algorithms use Chip-Seq data, which gives information about key SE marks (H3K4me1, H3K27ac, Med1, Oct4, Sox2, Nanog, etc.) [6] and is extremely helpful for the search, but some of them take steps to move away from using ChIP-Seq data.

**Figure 2:**
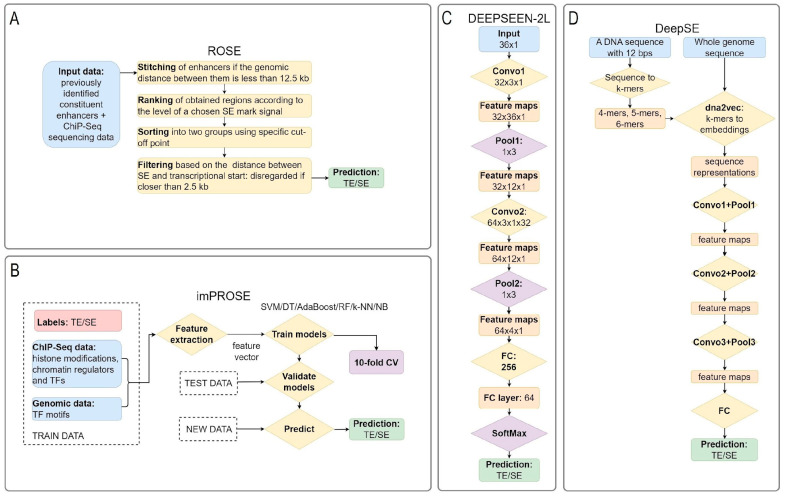
Currently implemented models for predicting super-enhancers. (**A**) ROSE; (**B**) imPROSE; (**C**) DEEPSEEN; (**D**) DeepSE.

At the moment, the most-used tool and the gold standard for solving this problem is the linear ROSE algorithm [3,9]. The main idea of the algorithm is to stitch enhancers if the distance between them is less than the threshold value (12.5 kb) and to rank them based on the level of SE marks’ signal from the Chip-Seq data (Figure 2A). Other tools that do not use ML methods have not gained popularity.

Aziz Khan and Xuegong Zhang performed ML analysis of the importance of SE features and developed the imPROSE tool (integrated methods for prediction of super-enhancers) based on ML algorithms to predict which enhancers are SEs [32]. An analysis of ChIP-seq data, RNA-seq data, Gene Ontology (GO), and DNA motifs was performed. The aim was to find a minimal subset to differentiate SE and TE. They used a random-forest-based approach, Boruta [33], for feature importance ranking and found that Med1, Med12, H3K27ac, Brd4, Cdk8, Cdk9, p300, and Smad3 are more informative for the task. The top six features according to their research are Brd4, H3K27ac, Cdk8, Cdk9, Med12, and p300. Then, they implemented six classical ML models, including Random Forest, linear SVM, k-NN, AdaBoost, Naive Bayes, and Decision Tree, and they performed 10-fold cross-validation and compared them (Figure 2B). The best results were shown by the Random Forest (AUC = 0.98). While using only DNA sequence features, such as conservation scores (phastCons), GC content, and repeat fractions, the authors achieved the AUC = 0.81. This shows that only DNA sequence characteristics can be used to distinguish TEs and SEs. Compared with ROSE, a H3K27ac-based method, imPROSE trained on Smad3 and H3K27ac data makes more predictions for cell-type-specific SEs in pro-B cells.

The imPROSE source code is available on GitHub and can be used to choose the model and its features, train the model, and make predictions. The input is a CSV file with the computed available features for the enhancers. The output is also a CSV file with the field Class and the class flag (SE or TE).

With the development of the NN approaches came the idea to use them for SE prediction. There are some works offering convolutional neural networks (CNN) to perform this task. DEEPSEN, a CNN implemented in 2019, was the first NN model used for this purpose, and it was implemented on TensorFlow with Python [34]. At that time, it outperformed all existing methods. The authors tested CNNs with different numbers of convolutional layers (from 2 to 4—DEEPSEN-2L, DEEPSEN-3L, and DEEPSEN-4L) and two fully connected layers, and they selected 36 features (Figure 2C). Each convolution layer included two steps: a convolution step and a pooling step. They used ReLU as the activation function, Adam optimization, and the cross-entropy loss function. The code is also available on GitHub, and the input data structure is similar to that of imPROSE.

The next step was implementing DeepSE in 2021 by Keras [35]. It outperformed methods that existed at that time using only sequence feature embeddings. It was shown that it can be used for different cell lines, which suggests that cell-specific SEs may share hidden sequence patterns. The researchers collected data from a number of human and mouse cell types. The novel moment was using the k-mer sequence embeddings obtained by training dna2vec on genomes. Then, the CNN classifier with two convolutional and two fully connected layers (Figure 2D) performs the binary classification (TE or SE). The code is available on GitHub. The first step of the pipeline is encoding the sequence for the particular cell type, and the second is choosing a model, training it, and making predictions.

Nowadays, neural network methods are undergoing a period of active development, and new approaches are emerging for working with sequences, in particular, biological ones. Their potential applications for in silico searches for super-enhancers look promising.

#### 2.2.2. Experimental Validation and Characterization of Super-Enhancers

To validate the predictions of super-enhancers with computational methods, it is necessary to carry out a number of experiments. Here, we consider experimental approaches to the study.

Much of the experimental research on super-enhancers involves the use of various genome-wide, sequencing-based approaches. They make it possible to identify SEs by their characteristic marks. Like typical enhancers, super-enhancers are characterized by increased sensitivity to DNase I, which is common for active chromatin structures. The results are cell type-specific [36,37]. This makes DNase-seq [38] a powerful and widely used approach in the search for and characterization of TE and SE. The identification of other marks is possible using ChIP-seq, a DNA–protein interaction characterization method. It makes it possible to detect common SE nucleosome architecture and TF binding sites [39,40,41]. SE ChIP-seq studies focus on such marks as H3K4me1, H3K27ac, p300, Med1, BRD4, LSD1, etc. [3,42,43,44]. DNase-seq and ChIP-seq are the major techniques for SE identification, but there are some other tools that can be used for this purpose. ATAC-seq, a technique used to assess genome-wide chromatin accessibility for transposase, has such advantages as high sensitivity and superior procedure speed [45]. It is also used in SE research [19,46,47,48]. Another chromatin accessibility estimation method, FAIRE-seq, which is based on formaldehyde crosslinking in vivo [49], is an alternative to DNase-seq and ATAC-seq, but it is sometimes used for SE validation [50]. There are some methods to measure noncoding RNA, including eRNA, such as GRO-seq [51,52]. It is another potential way to search for SEs and study their biology [53,54].

Many authors study the functioning of super-enhancers by subjecting them to certain manipulations, including DNA editing, the downregulation of certain genetic elements, or the inhibition of proteins closely connected with SE functioning. Deletions of SE or some of their parts help to estimate their role in epigenetic regulation and are performed using, for example, in vitro or in vivo CRISPR/Cas editing [46,55,56,57,58,59] or Cre/Lox recombination [55]. The results of such manipulations are measured using the aforementioned techniques or through the estimation of expression levels at the RNA level (microarrays, qRT-PCR, RNA-seq, etc.) or at the protein level (reporter assays, ELISA, Western blot, flow cytometry, etc.). If the goal is to have an influence on cancer cells, some features such as microscopy techniques, cell proliferation, and migration assays can measure cell metabolic activity (for example, the MTT test).

Another interesting and, in recent years, actively developed direction in the study of super-enhancers is their study in the context of the three-dimensional organization of the genome. Chromatin interaction measurement technologies (3C, 4C, 5C, Hi-C, and NG Capture-C) are used for this purpose. Often, methods of measuring expression are connected to the study of this topic, as the combination of these techniques makes it possible to establish correlations between changes in the structural organization of the genome in different cell types or after a certain type of exposure and levels of gene expression. These studies are aimed at finding answers to questions about how SEs are organized in space and what effect they can have on the expression of distant but spatially close genes.

### 2.3. Three-Dimensional Organization of Super-Enhancers

The functionality of eukaryotic genomes is mostly determined by their 3D organization. Understanding the stochastic expression of large gene cluster subsets dependent on chromatin contacts and 3D regulation has made significant progress [60,61,62]. Methods for measuring physical proximity between genomic sequences in fixed cells, known as chromosome conformation capture (3C), revealed that chromosomal contacts are organized into submegabase domains of preferentially insulated interactions known as topologically associating domains (TADs).

TADs are primarily formed by nested interactions between convergently oriented binding sites of the DNA-binding protein CTCF, which are established as chromatin-bound CTCF that arrest the loop-extruding cohesion complex [63]. Allelic insulation via CCCTC-binding factor (CTCF)-mediated directional looping may be epigenetically regulated by CTCF-binding site (CBS) element methylation [64,65]. CTCF is an eleven-zinc finger (ZF) multivalent regulator of transcription that recognizes numerous motifs using different combinations of its ZFs. It can act as a transcriptional activator, repressor, or insulator protein, thereby preventing enhancers and promoters from communicating. The CTCF protein binds to several thousand genomic loci in a given cell type, the vast majority of which are intergenic, and a subset of these sites overlap with transcriptional enhancers. While bound to chromatin domain boundaries, CTCF can recruit other TFs. CTCF-binding site mapping in various species has revealed that CBS elements are found throughout the genome. CTCF has the ability to directly affect transcription at promoters as well as to orchestrate interactions between regulatory elements and help separate eu- and heterochromatic areas in the genome at more distal sites by acting as a chromatin barrier. For example, in mouse thymocytes, the binding of CTCF and cohesin is highly enriched at enhancer and SE sequences, and cohesin itself facilitates spatial enhancer clustering, according to local and global 3C analyses [66]. Another study expanded the scope of SE functional reach beyond its respective target and past several CTCF sites into a juxtaposed neighborhood. Thus, mutational analysis demonstrated that the *Wap* SE controls Ramp3 (which plays an important role in cancer development), despite three separating CBSs. Deleting all of them resulted in the elevated expression of Ramp3 in mammary tissue, whereas deleting one distal CBS lowered Ramp3 expression in non-mammary tissues. Although these CTCF sites, including the loop anchor, do not prevent the super-enhancer from being activated, their absence from the mouse genome demonstrates their ability to suppress gene activation [67]. The idea is supported by the data obtained in the study of the Prdm14 SE in mouse embryonic stem cells (mESCs). The enhancer insulation proved to not be solely dependent on loop formation between its flanking boundaries. It was also shown that the SE activated the *Slco5a1* gene beyond its prominent domain boundary. Thus, the loop extrusion model is complemented by the fact that cohesin loading and extrusion trajectories originate at an enhancer contribute to gene activation [68]. Additionally, computational simulation in silico and genetic deletion in vivo revealed that tandem-arrayed CBS elements ensure balanced usage of associated promoters in specific and equal spatial chromatin contacts in general. Thus, studies of *Pcdh*, *β-globin*, and *Igh* SE clusters in HEC-1-B, K562, and Neuro-2A cells suggested that directional CTCF chromatin looping between convergent CBS elements underlies insulator function and accessibility of the tandem CTCF sites’ balanced promoters in 3D genome folding and regulation [55,69]. Recent research indicates that the transcription apparatus is compartmentalized and concentrated at SEs [70,71], resulting in phase-separated condensates that drive the expression of cell identity genes. Recently, it was demonstrated that CTCF is required for RNA polymerase II (Pol II)-mediated chromatin interactions at SEs, which appear as hyperconnected spatial clusters. Moreover, CTCF clustering is independent of liquid–liquid phase separation (LLPS) and is resistant to transcriptional perturbation, and it might have an instructive role and act as an architectural prerequisite in the formation of transcriptional condensates [72]. More detailed studying of CBS sites clustering reveals the existence of so-called “persistent” or constitutive CTCF sites for eight different cell types (GM12878, K562, Hela, IMR90, HUVEC, NHEK, HMEC, and KBM7), which are enriched at TAD boundaries. The deletion via CRISPR-Cas9 of two persistent CTCF sites at the boundary between a long-range epigenetically active (LREA) and a silenced (LRES) region within the Kallikrein (KLK) locus—a deregulated region of interest in prostate cancer—resulted in the concordant activation of all eight KLK genes within the LRES region.

CTCF genome-wide depletion alters TAD structure (including TADs merging), meaning that higher-order chromatin structures are supported by groups of essential constitutive CBSs [73].

Another TF mediating SE spatial activity, Aire, is the driver of self-reactive thymocyte clonal deletion and the generation of perinatal regulatory T cells. Aire controls immunological tolerance by driving the promiscuous expression of a large swath of the genome in medullary thymic epithelial cells (mTECs). Thus, ex vivo Hi-C experiments on mTECs from murine thymi together with biochemical and in vivo loss-of-function analyses confirmed that Aire regulates chromatin looping by evicting CTCF from domain boundaries and favoring the accumulation of cohesin on super-enhancers [74].

### 2.4. SEs and Transcription Regulation in Different Cell Types

With the knowledge of constitutive CBSs, given that SEs have varying sensitivity to chemical inhibitors, as discussed later in this review, and given that super-enhancers of many genes act with functional redundancy [75], it is reasonable to assume that only specific groups of SEs have high sensitivity to perturbations. In this case, there is a certain subset of SEs that function constitutively, providing a powerful level of background transcription for highly expressed genes. One of the first authors who hypothesized and showed the constitutive expression of SEs was Jung and colleagues in 2019 [31], calling such SEs “common”. They analyzed 30 human cell and tissue types in total, including 5 H1 ESCs and their derivatives (mesendoderms, mesenchymal stem cells, neuronal progenitor cells, and trophoblasts), 8 immortalized cell lines (with a focus on colon cancer cell line HCT-116), and 17 postmortem tissue types stemming from the circular, digestive, endocrine, and other systems. So-called “super-enhancer domains” (with an average length of 32 Kb compared to 26 Kb in individual SEs) were defined with the ROSE algorithm based on H3K27ac signals. The genes associated with common SE domains showed universally high expression, 82.4% of which were non-housekeeping genes that were GO-enriched and showed increased cell motility, transcription, and cell proliferation regulation, thereby implying the existence of distinct biological functions within common SE domains. Common SE domains showed higher conservation rates and low context-dependent tolerance scores with a surprisingly low correlation with the genes in close proximity, which points at an additional biological role of these domains that, according to the authors, is related to the early establishment of 3D chromatin loops (20 min after cohesion recovery). The authors speculated on three possible models of the SE domain in chromatin organization: loop recovery acceleration via architectural protein recruitment, a sequential model, and a hierarchical model. However, the hypothesis needs further experimental validation, as the result was obtained only for the HCT-116 cell line.

Furthermore, constitutive SEs are described in another CRISPR-based work devoted to the study of SE landscape dynamics in cell differentiations of several lineages. The authors discovered that there are three distinct SE patterns (which are probably SE subtypes): conserved, temporally hierarchical, and de novo. The temporal order of establishment of elements within SEs may be guided by DNA sequences. It has been shown in differentiated cells that both early- and late-emerging enhancers are indispensable for target gene expression, whereas in undifferentiated cells, the early enhancers are capable of it [76].

A comparison of stretch enhancers with SEs revealed differences in active chromatin mark loading: SEs were mostly associated with H3K27ac and RNA Pol II and produced enhancer RNA (eRNA), whereas stretch enhancers were enriched with H3K27me3, depleted of H3K27ac, and did not produce eRNA [24]. In addition, SEs, despite overlapping with a small fraction of stretch enhancers, were more cell type-specific than stretch enhancers. In another study, so-called “differential SEs” were proposed via the computational method DASE, which helped to identify the internal dynamics by summarizing them. The authors categorized differential SEs into five major groups based on their overall activity and structural alterations: overall-change, shortened, hollowed, shifted, and others, which allowed them to link SEs with different numbers of genes and assess their gene expressions’ divergent impacts. Besides that, DASE demonstrated the increased power of identifying cell line-specific SE regulation when applied to similar cell lines [77].

When talking about spatiotemporal SE dynamics, it is important to understand the possible variety of modes of interaction between SEs and their constituent parts. For instance, with the help of an integrated approach including ChIP-seq, scRNA-seq, and scATAC-seq data, the murine retinal SE Vsx2 double modality was shown, which reflected distinct developmental stages and cell type activity in vivo [46]. The CapStarr-seq high-throughput method developed for accurate quantification of enhancer activity has helped to identify associations between tissue-specific TF binding complexity and SEs [78]. The enhancer × promoter self-transcribing active regulatory region sequencing (ExP STARR-seq) approach allows for the systematic quantification of enhancer–promoter compatibility in humans, which was shown in K562 cells. The method identifies two classes of enhancers and promoters that exhibit subtle preferential effects. Housekeeping gene promoters contain activating motifs for factors such as GABPA and YY1, which reduce promoter responsiveness to distal enhancers. Promoters of variable-expression genes lack these motifs and respond more strongly to enhancers [79]. Additionally, dissection/reinsertion experiments with well-characterized SEs, such as the erythroid α-globin SE [80,81], would aid in revising current models by which SEs are thought to contact and activate their cognate genes.

### 2.5. SE Conservation in Humans, Mice, and Other Placental Organisms

One of the pending questions about enhancers and SEs in particular is deciphering their evolution and conservation history. It seems quite complicated to show how a single enhancer/SE can be conserved across the animal kingdom, but enhancers and SEs may be as ancient and conserved as the TFs they interact with.

However, a study by Wong et al. demonstrated that in zebrafish and mouse embryos, putative enhancers identified in sponge *Amphimedon* sp. microsyntenic areas control patterns of cell type-specific gene expression. These sponge enhancers lie in the regions of microsynteny that are orthologous to those found in other metazoans and contain substantial histone H3K4me1 enhancer signals, even though they do not share considerable sequence identity with vertebrates [82]. Previous research found that some enhancers are conserved between humans and mice [83], whereas other enhancers may have been reprogrammed (RPE) as a result of human–mouse speciation [84] or during cancer progression [85,86].

Historically, putative enhancer sequences in humans with higher rates of evolution are associated with so-called human accelerated regions (HARs). These HAR sequences are thought to be responsible for human-specific phenotypic adaptations, as shown with the brain developmental transcription factor neuronal PAS domain-containing protein 3 (*NPAS3*) [87]. Along with other putative human-specific enhancers discovered in genome-wide surveys, HARs provide a rich dataset for the discovery of the unique gene regulation characteristics that distinguish humans, as it was demonstrated for a set of heart tissue-associated enhancers [88]. Another example is human accelerated non-coding sequence 1 (*HACNS1*), a positively selected limb enhancer sequence showing evidence of accelerated evolution and acquiring novel TF-binding sites in the human lineage compared to other vertebrates [89]. Transgenic mice with human *HACNS1* show increased transcriptional activity compared with the chimpanzee and macaque orthologous enhancers, which is probably due to mutations that prevent repressor binding to the gene in a distinct 81-bp region [90]. Data on noncoding HARs combined with genome-wide analyses of chromatin marks and TF/cofactor binding as well as further experiments on a set of human and chimp enhancer orthologues reveal that the vast majority of enhancers, as well as SEs, can have functional activity in transgenic mice; however, their regulatory effects may differ from one species to another [91]. Emerging evidence suggests that SEs can act as master regulatory hubs in humans and mice, controlling cell identity [92] and pluripotency [93,94]. However, it is unclear whether pluripotency-associated SEs share a deeply common evolutionary origin in mammals.

Extensive comparative epigenomic and transcription factor binding analyses in pigs, humans, and mice proved that SEs evolve rapidly in mammals. In addition, 30 shared transcription factors and BRD4 were found to be conserved activators of mammalian pluripotency-associated SEs in parallel with three pluripotency-associated SEs (SE-SOX2, SE-PIM1, and SE-FGFR1) that are highly conserved in mammals [95]. In SE conservation studies, it is probably more accurate to speak of functional conservation and/or structural conservation in regard to the pool of regulated genes both in studying the evolution of enhancers in mammals [96,97] and in vertebrates in general when, for example, comparing the results to zebrafish [98]. Nevertheless, the genomic distribution of zebrafish TEs and SEs differs from that of mammalian regions. Generally, SEs are more cell- and tissue-specific than TEs and are associated with a conserved set of genes throughout vertebrate evolution. Authors also speculate that SEs are more effective in defining tissue identity than broad H3K4me3 domains [98].

Studying the evolution of SEs should help to correctly adapt animal models of diseases. Thus, in a characterization of cis-regulatory element functions in 12 diverse tissues from four pig breeds, using strategies similar to those used in ENCODE and the Roadmap Epigenomics projects (RNA-, ATAC-, and ChIP-seqs), more than 220,000 cis-regulatory elements were identified in the pig genome. Surprisingly, regulatory elements between human and pig genomes were found to have higher conservation rates than those between humans and mice [99]. Another study focused on the investigation of the *Nppa* and *Nppb* genes in the heart (encoding atrial and B-type natriuretic peptides, respectively) and identified an evolutionarily conserved SE required for the spatiotemporal expression pattern and stress induction of Nppa and Nppb [100]. Apart from that, the enhanced knowledge of SEs aids in the investigation of novel aspects of the classical molecular mechanisms of gene expression regulation, such as alternative splicing [101], and of more complex processes, such as DNA repair coupled with oncogenic SE activation [102] and eRNA production, implying its promoter-like activity [103,104]. Needless to say, SEs seem to be as ancient as the heat-shock (HSP) response and conserved not only in the placenta [105,106]. The stress-remodeled yeast nucleome was suggested to bear functional and structural resemblance to mammalian SEs and to be controlled by transcriptional condensates.

In addition, studies show that SEs have different levels of evolutionary conservation when they are unequivocally defined and calculated via different algorithms [2,107]. All methods demonstrate a higher overlap with conserved elements compared to random chance; however, the enhancers detected via eRNA transcription were the most conserved, whereas the enhancers obtained using PTM marks were the least conserved [107].

Taken together, SE evolution is dependent on the organism and cell type; the inability to identify conserved SEs appears to be due to their ability to evolve faster than both the TFs with which they interact and the genes which they regulate. The terms defining SEs are ambiguous and requires a complement of experimentally validated facts. To achieve robust and reproducible results, a better understanding of how the various enhancer identification strategies in use today relate to the dynamic activity of gene regulatory regions is required, along with improvements to experimental methods and data analysis and the possible implementation of machine learning approaches [108]. As soon as the unity of nature and knowledge can be effectively proved “from the contrary”, it is important to acknowledge the necessity of the SE concept regardless of whether we want to disprove or prove the existence of super-enhancers as a distinct regulatory element.

## 3. Biological Role of Super-Enhancers in the Development of Diseases and Healthy Tissues

### 3.1. The Role of Super-Enhancers in Non-Pathological Processes

Super-enhancers were described as regulators in the fields of stem cell biology, differentiation, and cell-specific gene expression [5]. In this section, we describe the role of SEs during the ontogenesis and growth of healthy tissues.

It is known that maintenance of cell pluripotency requires a huge and complex regulatory network that includes a large number of transcription factors [109,110]. Super-enhancers were described in studies of mouse embryonic stem cells (mESCs)’ pluripotency maintenance. The main TFs, namely Oct4, Sox2, Nanog, Klf4, and Esrrb, were shown to occupy unusual enhancer domains, which were later called super-enhancers [3]. These transcriptional factors recruited the Mediator complex to activate the genes responsible for mESC pluripotency. The majority of the analyzed SE-controlled genes encoded such TFs as Oct4, Sox2, and Nanog and thereby formed an autoregulatory expression network. It was also shown that SEs control some other genes associated with pluripotency, such as coactivators, chromatin regulators, and shRNA [3]. There are additional TFs (Nr5a2, Prdm14, Tcfcp2l1, Smad3, Stat3, and Tcf3) that also occupy SEs and contribute to super-enhancer and expression stability [5]. One of the proposed mechanisms of Oct4/Sox2/Nanog (OSN) is the regulation of the pluripotency described through OSN recruitment via Ash2l followed by Ash2l/OSN complex formation at Nanog, Sox2, Oct4, and Jarid2 super-enhancers [111]. Interestingly, *Sox2* super-enhancers are responsible for more than 90% of Sox2 expression, and *Sox2* seems to be its only target. The knockout of *Sox2* SE caused dramatic changes in mESCs morphology and proliferation, as Sox2 is among the key TFs associated with pluripotency [56,112]. Nanog expression could be regulated by three super-enhancers, −45, −5, and +60, where downstream SEs (−45 and −5 kb) have stronger effects on Nanog expression. Dppa3 expression is also regulated by −45 SE, which provides transcriptional repression and chromatin condensation during the production of functional oocytes [113]. Enhancer RNAs produced during SE transcription also take part in its interactions with the *Dppa3* promoter region [114]. The obtained data implies that *Nanog* SEs do not have the same effect on cell fate. Supposedly, this feature is explained by the existence of redundancy among enhancer elements. For example, one could become fully functional only if both the −5 SE and −45 SE are deactivated [88]. For both mESCs and human ESCs (hESCs), BRD4, a member of the BET bromodomain family and a transcriptional regulator, was shown to play an important role in pluripotency maintenance. Its inhibition led to expression changes of genes associated with the identity of ESCs. To determine the mechanism of BRD4 action, ChIP-seq experiments were performed. These experiments showed that BRD4 occupies SEs and has a strong effect on their activity. The level of SE-controlled gene expression relies on the binding of Med-containing complexes, which is also BRD4-dependent [115]. Spt6 is a histone chaperone that disassembles and reassembles H3-H4 dimers [116]. It was shown that *Spt6* depletion leads to the expression downregulation of the pluripotency maintenance genes, as ESC super-enhancers show high enrichment levels of Spt6. In that study, Spt6 controlled the ratio between H3K27 acetylation and methylation [117], which confirmed the importance of super-enhancers in stem cell biology and epigenetics. Finally, it seems important that there are super-enhancers connected with pluripotency and are highly conserved in mammals. It was shown that the disruption of super-enhancers which regulate SOX2, PIM1, and FGFR1 expression leads to the loss of stem cell pluripotency [118].

Cell- and tissue-specific human SEs are also a common research focus. For example, five (NFIL3, KLF15, RXRA, SNAI2, and BCL6) and three (MEF2A, FLI1, and ETS1) SEs target TFs that are associated with adipocyte-selective and osteoblast-selective SEs, respectively, forming core regulatory circuitry (CRC, which is a group of interconnected auto-regulating TF-forming loops able to bind to not only their own SEs but also the SEs of other TFs within the loop). Furthermore, the findings show that osteoblast-selective SEs pre-exist in hMSCs, whereas adipocyte-selective SEs are generated only after adipocyte induction [119]. In a similar fashion, SEs stimulate osteogenesis in human bone marrow mesenchymal stem cells (hBMSCs). KEGG analysis of SE target genes before and after osteogenic differentiation showed that the TGF-β, PI3K-Akt, and ECM receptor signaling pathways are highly enriched TFs within SEs, and therefore, they are closely related to osteogenic differentiation [120]. Two lines of stem cells, mESCs and mouse epiblast stem cells (mEpiSCs), have different SE profiles. A number of SEs become activated in primed pluripotency and stay active in somatic derivatives in contrast with naïve cell lines [121]. However, SEs are not only involved in embryoblast regulatory networks. It was shown that super-enhancers in trophoblast stem cells (TSCs) have an influence on genes that control trophectoderm lineage development and placentation. Trophectoderm-specific master TFs (Gata3, Tead4, and Tfap2c) bind to SEs that control DNA-binding TFs and factors involved in signaling pathways (PI3K-Akt, Hippo, MAPK, etc.) [122]. In addition, SEs control some aspects of the primary germ layers’ differentiation process within triploblastic animals. For ectoderms, for example, corneal epithelial development is controlled not only by TFs but by 1154 SEs as well. These super-enhancers are loaded with ETS transcription factor family members (AP1, KLF, etc.). They affect the expression of genes such as PAX6, WNT7A, and MIR205HG, which are important for corneal epithelium formation [123]. Another example is CRC-SE, which can be found upstream of the *Vsx2* gene and is important for appropriate retinal development. There are Zfhx3, Prox1, Vsx2, Dbp, Hlf, Otx2, Isl1, and Lhx4 binding sites, and four of them (Isl1, Lhx4, Prox1, and Vsx2) showed reduced expression if the SE was experimentally disrupted. The direct CRISPR/cas9 deletion of some enhancer elements led to SE malfunction and further developmental issues such as eye or retinal size reduction [46]. Regarding the mesodermal germ layer, there are many examples of SEs that can take part in cell differentiation. We combined the results of experimental research groups into a summary table (Table 2).

Endodermal germ layer differentiation and functionality also reveal a strong association with a group of SEs. In *Xenopus tropicalis*, endoderm activation-specific TFs (Otx1, Vegt, and Foxh1) bind to super-enhancers located near the endodermal cell fate genes (*pnhd*, *foxa2*, *foxa4*, *sox17*, *frzb*, *gata6*, *hhex*, and *admp*), thereby activating the target gene’s expression [133].

In adult organisms, SEs are also involved in tissue-specific cell differentiation. For example, regulatory T cells become a functionally mature subpopulation only under the control of the Treg super-enhancers. These SEs are activated by Satb1 and regulate the *Foxp3*, *Il2ra*, and *Ctla4* Treg cell signature genes [134]. There are a number of well-studied tissue-specific super-enhancers. Some of these studies aid in understanding the mechanisms of SE functionality. In vivo experiments performed on murine models were conducted to study the *α-globin* super-enhancer. Four enhancer elements within this SE (R1-4) showed high Med-1 signals and were evolutionarily conserved. Deletions of these parts showed that only two of them (R1 and R2) had a significant effect on erythropoiesis in vivo. No synergic effect was observed; therefore, it seems that enhancers within SE act independently and mostly in an additive manner. The authors suggested considering such cases as individual enhancers rather than as higher-order structures [19]. A question arose on the individual enhancer elements’ roles within SEs, and this was also studied in Ly6Clow monocytes. The TFr Nr4a1 is the master regulator characteristic for the SE, which has three conserved parts: E2, E6, and E9. Only E2 was shown to be essential for Ly6Clow monocyte development and cell growth. It is possible that E6 and E9 can regulate Nr4a1 expression in some other conditions, but their primary effect and importance are not yet clear [135]. There is a complex experimental study dedicated to the role of super-enhancers during mammary gland cells’ ontogenesis. Here, researchers paid special attention to the Wap-associated SE. Whey acidic proteins (WAP) are highly expressed in mammary tissue and are activated during pregnancy. The *Wap* SE consists of three enhancer elements (E1, E2, and E3). At mid-pregnancy only E1 is fully occupied by the regulators, but between the 14th and 16th days of pregnancy, E2 and E3 are also activated. This leads to a 100-fold increase in Wap expression. The studied mutagenesis of E1, E2, and E3 enhancer elements revealed significantly different in vivo effects; that of E3 was the most influential. E2 and E3 had additive interactions. The simultaneous inactivation of all three enhancers caused a 1000-fold decrease of Wap expression. That study, in spite of the additive model, testified to the existence of an inner hierarchy of enhancer elements within the SE [136]. Further research on this super-enhancer showed that E1 is not required for E2 and E3 activation at terminal differentiation stages during lactation. In addition, when transferred to permissive chromatin, *Wap* SE retained mammary specificity, which indicates the weak regulatory potential of chromatin in mammary gland cell specificity [137]. Super-enhancers also control neuroplasticity, as the NMDA response involves SE reorganization and is associated with genes of neuronal identity (*Ncam1*, *Mapt*, *Rbfox3*, etc.) and neuronal activity (*Fos*, *Per1*, *Ephb2*, etc.) [50]. With the help of the rank ordering of SEs, epigenetic rewriting, and enhancer deletion analysis, renin cells, crucial for survival and homeostasis, were found to harbor a unique set of SEs that determine their identity, including the classical renin enhancer and *Rbpj*-enhancer. That study compared renin-phenotype cells at different physiological states. Comparing normal unstressed JG cells, chronically recruited renin-null cells, acutely recruited cells from mice subjected to sodium depletion plus captopril, and constitutively active As4.1 cells a notably reproducible pattern of open chromatin along via ATAC-Seq was revealed [138].

Interesting patterns may be associated with eRNA. eRNA expression and stability is considered not to be strong enough for regulation in trans. The higher transcriptional activity of TE compared with SE suggests that some of them could produce the non-coding RNA involved in trans-regulation. For example, alncRNA-EC7 (or Bloodlinc), which is transcribed from a SE needed for the expression of BAND3/SLC4A1, an erythroid membrane transporter, is also trans-acting and regulates about 500 target genes. Bloodlinc also interacts with TFs and chromatin-organizing factors [139].

The use of SEs for cell identity profiling and prediction may be limited in terms of sensitivity and specificity, and it needs to be complemented with promoter-centric data when analyzing gene clusters [140].

Endodermal germ layer differentiation and functionality also show a strong association with group of SEs. In *Xenopus tropicalis*, endoderm activation-specific TFs (Otx1, Vegt, and Foxh1) bind to super-enhancers located near the endodermal cell fate genes (*pnhd*, *foxa2*, *foxa4*, *sox17*, *frzb*, *gata6*, *hhex*, *admp*), thereby activating the target gene’s expression [133].

### 3.2. Super-Enhancers and the Development of Diseases

Because super-enhancers take part in the control of such important processes as the maintenance of stem cell pluripotency, differentiation, and cell-specific gene expression, abnormal changes in their functioning can lead to pathological processes. It is known that there are SEs associated with tumorigenesis, developmental disorders, neurodegenerative processes, and some other diseases [5,26].

SEs are important regulators, and their activity modulation could cause pathological changes. Approximately 3000 SEs were shown to be cell-specific in microglia, neurons, and oligodendrocytes. A number of them harbored GWAS risk) variants for Alzheimer’s disease. Some GWAS variants were found within super-enhancers and affected Alzheimer-associated gene expression [141]. In addition, mice with Huntington’s disease had mainly decreased expressions of neuron-specific genes regulated by super-enhancers that were poorly enriched with RNAPII and H3K27ac histone modifications [142].

The research on super-enhancers aids in understanding and explaining aspects of complex inflammation mechanisms. SE-mediated transcription is an important part of inflammatory pathways and is connected with inflammatory disorders. NF-κB participates in the activation of endothelial enhancers and further proinflammatory activation and SE formation. These processes lead to global changes in the BRD4 landscape and the expression of endothelial proinflammatory factors such as TNF-α. Cytokine expression causes the rapid loss of non-inflammatory SEs, pushing cells and tissues into a pro-inflammatory metabolic state. In vivo BET bromodomain inhibition causes the suppression of atherogenesis [143]. A common mechanism was shown for human Simpson–Golabi–Behmel syndrome adipocytes. TNF stimulation is followed by the loss of cell-type specific SEs and formation of NF-κB-bound SEs. As a result, cell identity genes are repressed, and proinflammatory processes are activated [144]. It came out that the demethylation of H3K9 and H3K27 by demethylases KDM7A and UTX is important for NF-κB-signaling and the adhesion of endothelial cells during pro-inflammatory responses [145]. Th9 cell-mediated allergic inflammation is associated with the IL-9 SEs. The selective knockdown of BRD4 and Med1 along with inhibition via JQ1 all lead to SE disruption, anti-inflammatory IL-9 suppression, and, following Th9 differentiation, arrest under additional OX40 stimulation [146]. SEs associated with juvenile idiopathic arthritis are enriched by ETS- and RUNX1-binding motifs. JQ1 treatment has an influence on JIA-associated gene expression inhibiting disease-associated processes [147]. In some T-helpers (Th1, Th2, Th17) genes associated with cytokines and their receptors were mainly linked to super-enhancers. A number of immune-related diseases are connected with variations in *BACH2* loci under SE regulation. In addition, it was shown that cytokine signaling blockers, which are clinically effective in autoimmune diseases, predominantly affect genes associated with SEs [23]. A differentially methylated region (DMRs) is a region in the genome that has different methylation patterns among samples [148]. For atherosclerosis, it was estimated that hypermethylated DMRs often overlap super-enhancers. After the vascular smooth muscle cells transdifferentiate, the disease-associated and differentially methylated regions are common entities found within SEs characteristic of tissue-specific monocyte cells. In addition, elastin, whose expression is relevant to atherosclerosis, is significantly downregulated due to aorta-specific SE hypermethylation [149].

Genome-wide association studies were held to study the role of SNPs in SE regions in type 2 diabetes. The set of genes and SNPs within them were strongly associated with type 2 diabetes through glucose homeostasis, the G-protein coupled signaling pathway, the WNT signaling pathway, the negative regulation of inflammatory response, the positive regulation of lipid metabolism, etc. [150].

It has been demonstrated that particular SNPs and insertions/deletions inside super-enhancers not only correlate with different genetic illnesses but also have a high correlation with oncogenic expression and cancer in general [5]. Super-enhancers are crucial for the development of hematological malignancies. According to a number of studies, chromosomal translocations are the most typical mutation associated with hematological malignancies and the leukemogenesis process. For instance, a specific translocation, t(6;8)(p21;q24), was discovered in a blastic plasmacytoid dendritic cell neoplasm. It led to a potent RUNX2 super-enhancer interaction with the MYC promoter, allowing both oncogenes to be expressed simultaneously [151]. An inversion connected to the overexpression of the EVI1 gene was discovered in another investigation. The distant GATA2 super-enhancer was able to relocate to the EVI1 upstream and boost its expression as a result of this translocation [152]. Furthermore, because of distant SEs’ translocation at the promoter region of these oncogenes, multiple myeloma is usually linked to MYC and MYB overexpression [153,154].

In addition, super-enhancers should be taken into account as a component of a complex pluripotent transcriptional regulatory network, according to some research that uses the Core Regulatory Circuitry (CRC) paradigm [155]. One of the most crucial elements of a specific cancer type’s CRC, once it has been established, is the interaction between its transcriptional factors and SEs, which ordinarily do not have such a regulating influence. In other words, it is an integrated auto-regulatory loop that forms this kind of SE. For instance, SEs that upregulate MYC, JUNB, and FOSL1 have shown high cross-binding to the SE areas of other TFs that are crucial for ESCC progression during the cell cycle [156,157,158,159]. The *OCA-B* oncogene is upregulated by BRD4-loaded super-enhancers that interact with *POU2AF1* loci and cause the development of diffuse large B-cell lymphoma (DLBCL). A total of 285 genes with the most BRD4-loaded super-enhancers showed considerable downregulation as well as a potent anti-DLBCL effect after DLBCL was treated with BRD4 inhibitors [160].

According to a study conducted by Betancur P. and colleagues, SEs significantly upregulate the CD47 enzyme, which is highly expressed in T-ALL and breast cancer cells. Namely, a collection of particular SEs responsible for CD47 expression were found in breast cancer cell lines (HER2 or ER+ PR+) and breast tumors (ER+ PR+). However, when several cancer cell lines were tested, the consistency of the evaluated SE sets did not continue to be the same. It was demonstrated that each type of malignant cell has a unique core of super-enhancers that upregulate a unique set of oncogenes. Human mammary epithelium and CD3+ T cells both displayed significantly lower levels of CD47 expression and varied SE-profiles from tumor cells when compared. Accordingly, SEs of CD47 discovered in cancer cells were accompanied by newly generated super-enhancers, increasing the likelihood of high CD47 expression. This was proven using H3K37ac analysis and the treatment results of BRD4 inhibitors. Additionally, decreased CD47 expression and increased phagocytosis frequency in MCF7 cells occurred after inhibiting the TNF pathway with infliximab monoclonal antibodies [161].

According to Chen H. and Liang H.’s work, the expression of super-enhancer RNA and CpG methylation both play crucial roles in the development of the TCGA melanoma cancer type and other malignancies. Super-enhancer areas (377 Mb) containing >300,000 eRNA loci were found and allowed to assess the nuances of SEs activation using RNA-seq data. The Cancer eRNA Atlas was constructed in order to organize and facilitate the usage of the collected data. This online resource [162] offers information on SEs’ eRNA expression profiles and methylation metrics for a variety of cancer samples as well as for normal cell lines in addition to annotations of the analyzed SEs. Analysis of the function of related SEs during the formation of ovarian tumors became critically essential after high levels of BRD4 TF expression became distinctive in ovarian cancer patients and were found to influence the success of therapy. CRISPRi, CRISPR-KO, and Hi-C were utilized by Kelly R. and colleagues to characterize the regulatory role of ovarian cancer super-enhancers. Among the 86 most active SEs identified using CRISPRi, SE60 and SE14, which were the first two candidates to modify key genes involved in the development of ovarian cancer, attracted particular attention. These two targets were chosen for additional CRISPR knockdown, a technique that made it possible to determine the role of SEs in the regulation of genes expressed during quiescence, metastasis, and invasion (e.g., *RAE1* and *EPHA2*). The acquired results were confirmed with Hi-C tests to support the typical local regulatory role of the investigated super-enhancers and to provide confidence in the functional gene annotation [163]. An earlier ovarian cancer study revealed a substantial decrease in ALDH1A1 expression following BET inhibitor administration (e.g., JQ1). The suppression mechanism was found to involve repressing its own seRNA production and SE disruption in response to BRD4 inhibition. The use of BET inhibitors may be a promising therapeutic approach when ALDH is linked to resistance and ovarian tumor relapse [164].

A specific super-enhancer had an unexpected regulatory role in the neck squamous cell carcinoma, according to a recent study by Wan Y. and colleagues (HNSCC). MiR-21-5p is a well-known oncomiR that has been demonstrated to encourage the development of malignant cancer [165]. NFI, SRF, p53, STAT3, AP-1, and other repressors or activators are abundant in the promoter region of the *MIR21* gene, according to several studies [166,167,168]. In this study, it was discovered that the AP-1 member FOSL1 upregulates the expression of MIR21. Further research showed that FOSL1 was enhanced in the newly created MIR21 super-enhancer. Because MIR21 expression significantly decreased after JQ1 therapy or FOSL1 expression knockdown in HNSCC cells [169], the FOSL1-reliant mir-21-p production hypothesis was verified.

Thus, super-enhancers are crucial regulatory elements because they modulate cell fate during non-tumor pathological processes as well as those in cancerous cells. Several studies have been conducted in order to explain the concept of super-enhancers; other groups experimentally showed the role of SEs during various states.

## 4. Inhibitors of the SE-Mediated Transcription Positive Regulators

**Figure 3:**
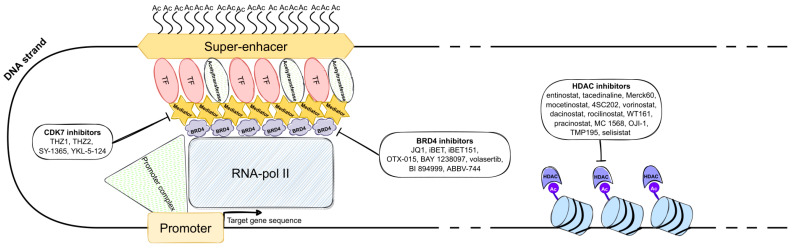
SE-regulated transcription inhibitors that can be used for cancer therapy.

The important role of super-enhancers in determining cell fate and development of various diseases, including tumors, suggests the idea of targeting super-enhancers’ regulatory elements, such as TFs and Co-TFs, during prospective therapy. Their inhibitors can be used both for research purposes, e.g., studying the biology of super-enhancers, and also for practical purposes, for example, the treatment of socially significant diseases. In this section, we review experimental works suggesting therapies for super-enhancers and their proposed inhibitors (Figure 3).

Because the goal is to block transcription, it is logical to influence the proteins that affect the function of RNAPII. That is why the most popular targets are BRD4, chromatin reader, and activator of RNAPII transcription at active chromatin marks [170], CDK7, which is a cyclin-dependent kinase involved in the regulation of RNAPII-activating phosphorylation [171,172]. Combination therapy is also a promising strategy that has gained popularity in recent years.

Here, we review SE inhibitors with different target proteins and different mechanisms of action. Some of them are SE-specific, whereas others have broad transcriptional effects but mainly affect SE function. These compounds have in common that they act on key elements involved in the formation and functioning of super-enhancers and therefore significantly change the super-enhancer profile. Next, we consider the experimentally studied groups of super-enhancer inhibitors and their specifics.

### 4.1. Selective Inhibitors of BET Bromodomains

Bromodomains (BRDs) are protein interaction modules that specifically recognize ε-N-lysine acetylation motifs. They play an important role in the reading process of epigenetic marks. BRDs are evolutionarily conserved and present in diverse nuclear proteins, including those in the BET family. BET proteins have two amino-terminal BRDs that bind to hyperacetylated promoter or enhancer regions [173]. Of particular interest in the context of super-enhancer research is BRD4, the best-characterized member of the BET family. It is one of the master regulators of SE [71] and one potential therapy target. BRD4, as well as Med1, can be used for SE annotation, and SEs are especially enriched with it [3,5,160]. It was shown that Med1 and BRD4 have an important role in the formation of condensates at SEs, which is described in the phase separation model [11,71]. Super-enhancers are particularly sensitive to therapy with these inhibitors [9]. The inhibitors of BET proteins (BETi) targeting one or both bromodomains can be an important step towards the goal of suppressing oncogenic networks in tumors. JQ1 is a well-known BET bromodomain inhibitor [174]. Although BRD4 is not only involved in the regulation of super-enhancers, it appears that it is controlled by super-enhancers that are particularly sensitive to JQ1 treatment [9]. Potentially, JQ1 can be used for the therapy of different diseases, including atherosclerosis [143], primary effusion lymphoma [175], skin cancers [176], ovarian cancer [164], colorectal cancer [177], osteosarcoma [178], breast cancer [179], brain tumor [180], prostate cancer [181], etc. JQ1 is not the only known BET inhibitor. I-BET, a benzodiazepine derivative, disrupts chromatin complexes responsible for the expression of key inflammatory genes in activated macrophages, and it confers protection against lipopolysaccharide-induced endotoxic shock and bacteria-induced sepsis [182]. Some BETi were shown to inhibit SE in tumors.

A number of researchers have shown that cancer cells can be resistant to BETi therapy. There are various causes of this resistance in different conditions. One reason can be BRD4 recruitment to chromatin in a bromodomain-independent manner [183]. Another mechanism of resistance is connected with the suppression of the PRC2 complex, which involves the activation and recruitment of WNT-signaling components to compensate for the loss of BRD4 and drive resistance in various cancer models [175]. For breast cancer, another mechanism was shown. PELI1 is upregulated in breast carcinomas, and it destabilizes LSD1. This leads to the decommissioning of the BRD4/LSD1/NuRD complex and the absence of the effect of JQ1 treatment [42]. There are other serious problems with JQ1 therapy. It was shown that it reversibly blocks IFN-γ production while IFN-γ is also regulated by SEs. This shows that BET inhibitors may disrupt the functions of the innate and adaptive immune response [184]. The possible strategies to overcome these difficulties include choosing other targets for therapy or combinational therapeutic targeting.

### 4.2. Selective CDK7 Inhibitors

CDK7 is a cyclin-dependent kinase that plays an important role in the regulation of RNAPII phosphorylation [171,172]. Targeting CDK7 is another way to reduce RNAPII-mediated gene transcription. SEs are enriched with the elements of transcriptional machinery and are downregulated with low concentrations. SE transcription is especially sensitive to CDK7 inhibitors [185,186,187,188].

The most popular CDK7 inhibitors are THZ1 [185] and THZ2 [189]. THZ1 is a phenylaminopyrimidine that targets a remote cysteine residue located outside of the canonical kinase domain. It is selective for CDK7 and shows an IC50 of less than 200 nM [185]. It was shown that CDK7 inhibition suppresses SE-linked transcription in MYCN-driven tumors [188]. There are a number of examples of using THZ1 for SE inhibition to prevent cancer progression. In some cases, for example, in mantle cell lymphoma and double-hit lymphoma, THZ1 helps to overcome tumor resistance to other drugs [190]. THZ2 is a modified THZ1 with altered regiochemistry of the acrylamide on THZ1 (from 4-acrylamide-benzamide to 3-acrylamide-benzamide). It also selectively targets CDK7 and has improved pharmacokinetic features and a fivefold improved half-life in vivo [189]. Like THZ1, THZ2 also attracted research as a potential SE inhibitor.

### 4.3. Histone Deacetylase and Demethylase Inhibitors

SEs are typically enriched in such post-translational modification histone marks as acetylation at H3 lysine 27 (H3K27ac) and mono-methylation at H3 lysine 4 (H3K4me1) [5]. This leads to the idea that effects on such histone modifications can affect the biology of super-enhancers, primarily their interaction with epigenetic readers. Thus, histone deacetylases and histone demethylases can become potential targets for inhibitors. Histone deacetylase inhibitors (HDACIs) can be rather effective. Because histone acetylation has an important epigenetic role, it is natural that HDAC inhibition leads to a wide range of effects and global responses. The inhibition leads to increased pausing of RNAPII and loss of H3K27ac at enhancers. It is important that HDACIs preferentially suppress SE-driven transcripts and cause the reduction of RNAPII signals on SEs [191].

For example, it was shown that the treatment of transformed cells with largazole leads to the remodeling of the enhancer structure by modulating H3K27ac in a dose dependent manner. It is also important that it preferentially suppresses SE-driven transcripts that are associated with oncogenic activities [191]. In rhabdomyosarcoma, hyperacetylated histones spread and disrupt the three-dimensional organization of SEs upon HDAC inhibition with entinostat. As a result, a decrease in the expression of SOX8, MYOD1, MYOG, and MYCN was observed [192]. HDAC1 and HDAC7 are also important for maintenance cancer stem cells (CSCs) in breast and ovarian tumors. Entinostat represses HDAC 1 and HDAC7 in BrCa cells with a stem-like phenotype, and the treatment reduces H3K27ac at stem cell transcription factor genes including *c-MYC*, *VDR*, *RB1*, *EZH2*, *c-JUN*, *HOXA2*, and *HOXA10* while increasing H3K27ac globally [193]. The effect of HDACi depends on the class of the target HDAC protein. HDACs are primarily divided by their dependence on either NAD (Class III) or Zinc (Class I, IIa/b, and IV) to catalyze deacetylation. Class I HDAC enzymes (HDAC1, 2, 3, and 8) are related to the regulation of transcription. Using *PAX3-FOXO1* fusion oncogene positive rhabdomyosarcoma as a model system and a panel of HDAC inhibitors with diverse and well-characterized isoform selectivity, it was shown that Class I HDAC inhibitors were the most effective, followed by Class IIa (HDAC4/5/7/9), Class IIb (HDAC6/10), Class III (SIRT proteins), and Class IV (HDAC 11) [194].

Another example is the dependence of PAX8, a prototype lineage-survival oncogene in epithelial ovarian cancer, on HDAC inhibition through the perturbation of the super-enhancer topology associated with the *PAX8* gene locus. Class I HDAC inhibitors such as panobinostat, romidepsin, and entinostat disrupt PAX8 transcription [195]. Panobinostat, romidepsin, and vorinostat can influence the Warburg effect by disrupting super-enhancers related to such genes as *MYC*, hexokinase 2 (*HK2*), *GAPDH*, and enolase 1 (*ENO1*) in glioblastoma models. These chemicals can be used to reprogram glioblastoma’s central carbon metabolism, which is controlled by SEs [196]. Among the histone demethylases, the most popular target is LSD1 (lysine-specific demethylase 1), which regulates gene expression by affecting histone modifications. Unlike the previously discussed inhibitors, LSD1 inhibitors do not suppress the work of super-enhancers, but instead remove LSD1 repression from them. For example, LSD1 inhibitors NCD25 and NCD38 inhibited the growth of MLL-AF9 leukemia as well as erythroleukemia, megakaryoblastic leukemia, and myelodysplastic syndromes. Activated super-enhancers regulate myeloid differentiation. The hematopoietic regulators that are abnormally silenced by LSD1 have anti-leukemic effects [197]. The activation of GFI1-SE during treatment with the same inhibitors leads to the differentiation of erythroleukemia cells [198]. Another example of demethylase inhibition is KDM6 histone demethylase inhibition by GSK-J4 in colorectal cancer. The treatment led to enhancers being reprogrammed, and SE-associated genes responded in a more sensitive manner. This weakened the malignant phenotypes of cancer cells [199].

### 4.4. Other Potential Inhibitors and Their Targets

In recent years, some key downstream molecules and pathways of SEs involved in various tumors have attracted more and more attention. New strategies for influencing super-enhancers are developing, and they have great potential.

Predominant downstream signaling pathways, for example, the MAPK/ERK pathway, play an important role in cell growth and proliferation for normal cell development and cancer progression [200]. The MEK kinase (MAP kinase kinase) is one of the key elements of this pathway. It phosphorylates and activates mitogen-activated protein kinase (MAPK). MEK is one of the most popular targets for MAPK/ERK pathway inhibition [201]. Influencing this metabolic pathway may lead to the remodeling of super-enhancers. It was shown that in rhabdomyosarcoma, MEK inhibition with trametinib causes the loss of ERK2 at the MYOG promoter as well as the transcriptional delay of MYOG expression. MYOG opens up the chromatin and establishes SEs at genes that are responsible for late myogenic differentiation [202,203]. Potent and selective MEK inhibitor AZD8330 affects glioblastoma stem cells’ viability at low micromolar doses through SE remodeling [204]. The JAK-STAT pathway also plays a huge role in cell growth, differentiation, apoptosis, and other integral cellular functions [205]. SEs are selectively targeted by a JAK (Janus kinase) inhibitor, tofacitinib, which can help in the treatment of immune mediated disorders, including rheumatoid arthritis [23]. Mediator-associated kinases cyclin-dependent kinase 8 (CDK8) and CDK19 prevent the increased activation of key super-enhancer-associated genes in acute myeloid leukemia (AML) cells. Cortistatin A, which inhibits Mediator kinases, has antitumor effects in vitro and in vivo [206]. Some of the drugs that have a potential influence on SE are specific to the tumor type. As an illustration, NR4A nuclear receptors are known to be tumor suppressors in AML. The drug dihydroergotamine (DHE) interacts with NR4A nuclear receptors and represses transcription of a subset of SE-associated leukemic oncogenes [207].

### 4.5. Combined Treatment Strategies

Combined therapy strategies have a complex effect on super-enhancers, and inhibitors in pairs can show synergies. In addition, the combination of inhibitors reduces the likelihood of drug resistance. One of the most popular combinations is that of BET inhibitors paired with CDK7 inhibitors. Such a therapy has shown increased efficiency in some tumor types.

BET bromodomain inhibitors are also combined with the inhibitors of other kinases. BRD4 is an important player in MYC SE transcription regulation, and cyclin-dependent kinases, especially CDK1 and CDK2, stabilize MYC phosphorylation. Suppression via the combined targeting of MYC expression and stabilization using BET bromodomain inhibition (JQ1) and CDK2 inhibition (milciclib), respectively, is a promising strategy to cure medulloblastoma [208]. BET bromodomain inhibitors were also used together with CREB-binding protein (CBP) inhibitors in diffuse intrinsic pontine glioma cells. CBP is an acetyltransferase associated with the acetylation of histones at SE. A combination of JQ1 (a BET bromodomain inhibitor) and ICG-001 (a CBP inhibitor) led to strong cytotoxic effects [209]. The repression of c-MYC is one important task in the therapy of many cancer types, including in colorectal cancer. Its overexpression due to hyperactive WNT/β-catenin/TCF signaling is one of the key phenomena for tumorigenesis. JQ1 suppresses c-MYC transcription, and trametinib, a MEK/ERK pathway inhibitor, affects post-translational mechanisms. The dual targeting of BET proteins and MAPK signaling shows synergetic effects as well [177]. In B-cell lymphoma, BET inhibitor OTX015 was combined with PI3Kα-selective inhibitors alpelisib and CYH33 and PI3Kδ-selective inhibitor idelalisib. CYH33’s performance together with OTX015 was especially successful and had such consequences as a decrease in acetylated H3 bound to the promoter and the super-enhancer region of *c-MYC*, the phosphorylation and proteasomal degradation of the histone acetyltransferase p300, and finally, cell cycle arrest and apoptosis [210]. It is known that patients with neuroblastoma with consequent N-Myc oncoprotein overexpression have a very poor prognosis. THZ1 suppresses super-enhancers regulated with the participation of CDK7, including the one that controls N-Myc transcription. Drugs such as ponatinib and lapatinib are tyrosine kinase inhibitors (TKIs). Tyrosine kinase performs c-Myc and N-Myc protein dephosphorylation and N-Myc stabilization. TKIs with THZ1 synergistically induce MYCN-amplified neuroblastoma cell apoptosis [211]. Another strategy is to co-target SEs with THZ1 and Bcl-2 or Bcl-xL, which are anti-apoptotic proteins, with BH3-mimetics (ABT263, WEHI-539, ABT199, etc.). This combination showed a strong cell-killing effect in glioblastoma [212]. HDAC inhibition, for example, with panobinostat, can be successfully combined with either JQ1 treatment (BET bromodomain inhibitor) or THZ1 (CDK7 inhibitor). In the case of diffuse intrinsic pontine glioma, both strategies were rather beneficial, and the pairs of inhibitors have shown synergy [213]. Moreover, panobinostat and THZ1 were also shown to be effective in neuroblastoma treatment, reducing JMJD6, E2F2, N-Myc, and c-Myc [214]. HDAC inhibitors, as mentioned before, influence the Warburg effect and reprogram the metabolism of glioblastoma cells. This resulted in the engagement of oxidative phosphorylation driven by elevated fatty acid oxidation (FAO). FAO inhibitors such as etomoxir influence lipid metabolism. A combination treatment with HDAC and FAO inhibitors led to animal survival in xenograft models [196].

## 5. Conclusions

In the current review, we discussed the current knowledge on experimental validation and prediction of super-enhancers (SEs), a topic of ongoing discussion in the scientific community. First, we dove into the emergence of the concept of “super-enhancers”. Despite little consensus on how to define SE regions, both in terms of their calculation and theoretical conception, super-enhancers are characterized as separate objects of research, comprising broad enhancer clusters with an exceptionally high degree of enrichment of transcriptional factors and of other gene expression regulators or chromatin marks, as determined with ChIP-seq, RNA-seq, ATAC-seq, etc., and they are historically associated with the increased expression of oncogenes and cell differentiation genes. Such a definition requires further clarification, especially when talking about so-called constitutive SEs, the nature of interactions between the SEs’ constituent parts, and alternative clusters such as stretch enhancers.

We also discussed the regulatory potential of super-enhancers, both in normal and pathological conditions such as oncological conditions, neurodegenerative (such as Alzheimer’s and Huntington’s) diseases, inflammatory processes, and others. The location of SE regions and the labelling of TADs as well as the annotation of CTCF binding sites suggest that they share a common nature of organization and interaction, which was confirmed in various studies using the DASE, CapStarr-seq, and ExP STARR-seq algorithms. A number of studies, including those that implemented CRISPR-Cas editing, have shown that SEs, due to their interaction with transcriptional factors, significantly increase the expression of target genes, making a huge contribution to cell differentiation, growth, development, and tumorigenesis.

There are some future research directions for SEs in diseases. First, it is still unclear which of the socially significant diseases, in addition to those for which they have already been well-studied and mentioned before, can be associated with changes in the functioning of super-enhancers. Second, the development of therapeutic approaches aimed at targeting SE may be quite promising. Given the potential importance of SEs in cell fate determination and disease development, we examined promising therapeutic avenues, including the use of inhibitors that target positive regulators of SE-mediated transcription. These include selective inhibitors of BET bromodomain-containing proteins and CDK7, histone deacetylase and demethylase inhibitors, and other potential targets and strategies for prospective combination therapies.

We provided an overview of bioinformatic methods used to search for SEs, with a focus on machine learning and experimental techniques that have been actively used in recent years. A separate task was to describe the methods of in silico SE prediction, with special attention to machine learning approaches, used for the discovery and prediction of these regulatory elements. The majority of these are based on Chip-Seq data and the use of convolutional (CNN) and recurrent (RNN) neural networks and their combinations, for example, the KEGRU network [215] and DNABERT architecture [216]. Similar algorithms would enable a better understanding of the super-enhancers’ organization and biological role in healthy cells and diseases, facilitating the development of effective treatment strategies.

Nevertheless, the existing techniques are not sufficient to facilitate complex approaches because modern algorithms ignore the chromatin structure, the probable lateral interactions of SEs and uncharacteristic target genes, the possible breakdown of super-enhancer insulation, hijacking mechanisms, and other non-canonic formation and decomposition mechanisms of super-enhancers. That is why in our future work, we aim to develop an approach that would allow us to take into account the maximum number of nuances that affect the formation of super-enhancers in tumors and try to resolve the existing uncertainty based on regulatory effect blurring.

The question about SE evolution remains open. Super-enhancers, which are historically associated with the human-enhanced region, are thought to be responsible for human-specific phenotypic adaptations when compared between mammals. At the same time, according to some data, there is reason to believe that super-enhancers can be much more ancient, if compared exempli gratia with stress response proteins in multicellular organisms. It should be noted that advancements in understanding the molecular mechanisms of diseases (particularly oncological diseases [217,218,219,220]) as well as the study of intermolecular interactions through high-performance optical methods [221,222,223], the ultrasensitive determination of biomolecules using magnetic nanomarkers [224,225,226,227] or label-free biosensors [228,229,230], and the processing of genome-wide data through high-performance methods such as cloud computing [231,232] may provide new opportunities for comprehending the role of SEs, potentially leading to the revision of their definition.

In conclusion, despite ongoing research, even if the definition of the object itself is currently incomplete, our understanding of SEs is evolving, and further experimental and computational studies are needed to fully comprehend their role in gene regulation and to further develop therapeutic strategies.

## Figures and Tables

**Table 1:** Definition of TEs and SEs and comparison of associated features.

Parameter	Typical Enhancer (TE)	Super-Enhancer (SE)
Length (Size)	~1500 bp on average	≤12.5 kb between constituent TEs, ten of kilobases long
Constitution	Detached region	Multiple enhancer regions—constituent TEs
Histone marks	H3K4me1—● * H3K4me3—∅ * H3K27ac—● *	H3K4me1—● * H3K4me3—? * H3K27ac—●●● *
Transcription complexes	Mostly bound by small amounts (usually—one) of TFs and Mediator	Reacher in TFs (BRD4, CDK7, p300, CBP) and Mediators, also bound by numerous co-regulators, might involve several RNAP II complexes
Relative transcriptional output	Mostly acting in cis Moderate transcription levels—●/●● *	Might be acting in trans as well as in cis Higher transcription levels—●●●● *
Bioinformatical identification approaches	ChIP-seq data analysis Gene expression annotation—GREAT, ChromHMM	ChIP-seq data analysis Gene expression annotation—GREAT, ChromHMM Identification by stitching algorithms—ROSE or its analogues, including DNABERT and other ML-alternatives (CNN, RNN)

* Special symbols: ●—minimum enrichment; ●●—moderate enrichment; ●●●—high enrichment; ●●●●—the highest enrichment; ∅—no enrichment; ?—needs further investigation.

**Table 2:** Progenitors of the mesodermal germ layer and its specific SEs.

SEs	Tissue/Cell Type	Key TFs	Target Genes	Refs.
340 SEs	Adipocytes in early adipogenesis	C/EBPβ, KLF4, KLF5, TIF-1β, GR, C/EBPδ, STAT1, STAT5A, JunB, FOSL2, ATF7, PBX1	Genes related with early adipogenesis (proliferation, extracellular matrix–receptor interactions, and cell growth)	[124]
BA SEs	Brown adipocytes	PPARγ, RXR, C/EBP, SIX1 (late-stage)	*Cebpa*, *Fabp4*, *Scd*, late stages: Ucp1, *Cidea*, *Fgf21*, *Ppara*, etc.	[125]
SLC25A37 SE	Erythroid cells	GATA1, TAL1	*SLC25A37* (encodes Mitoferrin 1)	[126]
543 SEs	Hematopoietic stem/progenitor cells	RUNX1, GATA2, FLI1	*ETV6*, *ERG*, *KIT*, *LMO2*, *MEIS1*, etc.	[127]
724 SEs	Chondrocytes	Sox6, Sox9	*Chst11*, *Col9a1*, *Wwp2*, *Acan, Sox9*, *Sox6*, *Runx2*, and *Fgfr3*	[128]
Cardiac SEs	Cardiac tissues	GATA4 and TBX5	Genes involved in cardiac development	[129]
2 SEs producing seRNA-1 and seRNA-2	Myocytes	MyoD, MyoG, TCF12, TCF3, MEF2D, PBX1, FoxO3	seRNA-1: myoglobin (*Mb*) and apolipoprotein L6 (*Apol6*); seRNA-2: *Atp1a1* and *Igsf3*	[130]
728 SEs	Osteoclasts	Irf8, Fli1, Mafb, Fosl1/2, Junb, Myc, Nfkb2, Spi1, Relb	TFs such as *Irf8* and *Fos*	[131]
784 SEs	Lung fibroblasts	TBX2, TBX4, TBX5, HOXA5, FOXL1, FOXP1, MEIS1, TGIF1	Genes associated with response to hypoxia and extracellular matrix organization	[132]

## References

[1] Pennacchio L.A., Bickmore W., Dean A., Nobrega M.A., Bejerano G. (2013). Enhancers: Five Essential Questions. Nat. Rev. Genet..

[2] Blobel G.A., Higgs D.R., Mitchell J.A., Notani D., Young R.A. (2021). Testing the Super-Enhancer Concept. Nat. Rev. Genet..

[3] Whyte W.A., Orlando D.A., Hnisz D., Abraham B.J., Lin C.Y., Kagey M.H., Rahl P.B., Lee T.I., Young R.A. (2013). Master Transcription Factors and Mediator Establish Super-Enhancers at Key Cell Identity Genes. Cell.

[4] Parker S.C.J., Stitzel M.L., Taylor D.L., Orozco J.M., Erdos M.R., Akiyama J.A., van Bueren K.L., Chines P.S., Narisu N., NISC Comparative Sequencing Program (2013). Chromatin Stretch Enhancer States Drive Cell-Specific Gene Regulation and Harbor Human Disease Risk Variants. Proc. Natl. Acad. Sci. USA.

[5] Hnisz D., Abraham B.J., Lee T.I., Lau A., Saint-André V., Sigova A.A., Hoke H.A., Young R.A. (2013). Super-Enhancers in the Control of Cell Identity and Disease. Cell.

[6] Pott S., Lieb J.D. (2015). What Are Super-Enhancers?. Nat. Genet..

[7] The ENCODE Project Consortium (2012). An Integrated Encyclopedia of DNA Elements in the Human Genome. Nature.

[8] Chen Y., Yao B., Zhu Z., Yi Y., Lin X., Zhang Z., Shen G. (2004). A Constitutive Super-Enhancer: Homologous Region 3 of Bombyx Mori Nucleopolyhedrovirus. Biochem. Biophys. Res. Commun..

[9] Lovén J., Hoke H.A., Lin C.Y., Lau A., Orlando D.A., Vakoc C.R., Bradner J.E., Lee T.I., Young R.A. (2013). Selective Inhibition of Tumor Oncogenes by Disruption of Super-Enhancers. Cell.

[10] Sengupta S., George R.E. (2017). Super-Enhancer-Driven Transcriptional Dependencies in Cancer. Trends Cancer.

[11] Hnisz D., Shrinivas K., Young R.A., Chakraborty A.K., Sharp P.A. (2017). A Phase Separation Model for Transcriptional Control. Cell.

[12] Bruter A.V., Rodionova M.D., Varlamova E.A., Shtil A.A. (2021). Super-Enhancers in the Regulation of Gene Transcription: General Aspects and Antitumor Targets. Acta Nat..

[13] Kundaje A., Meuleman W., Ernst J., Bilenky M., Yen A., Heravi-Moussavi A., Kheradpour P., Zhang Z., Wang J., Roadmap Epigenomics Consortium (2015). Integrative Analysis of 111 Reference Human Epigenomes. Nature.

[14] Niu D.-K., Jiang L. (2013). Can ENCODE Tell Us How Much Junk DNA We Carry in Our Genome?. Biochem. Biophys. Res. Commun..

[15] Doolittle W.F. (2013). Is Junk DNA Bunk? A Critique of ENCODE. Proc. Natl. Acad. Sci. USA.

[16] Eddy S.R. (2013). The ENCODE Project: Missteps Overshadowing a Success. Curr. Biol..

[17] Kellis M., Wold B., Snyder M.P., Bernstein B.E., Kundaje A., Marinov G.K., Ward L.D., Birney E., Crawford G.E., Dekker J. (2014). Defining Functional DNA Elements in the Human Genome. Proc. Natl. Acad. Sci. USA.

[18] Zhang T., Zhang Z., Dong Q., Xiong J., Zhu B. (2020). Histone H3K27 Acetylation Is Dispensable for Enhancer Activity in Mouse Embryonic Stem Cells. Genome Biol..

[19] Hay D., Hughes J.R., Babbs C., Davies J.O.J., Graham B.J., Hanssen L.L.P., Kassouf M.T., Oudelaar A.M., Sharpe J.A., Suciu M.C. (2016). Genetic Dissection of the α-Globin Super-Enhancer in Vivo. Nat. Genet..

[20] Schulz V.P., Lezon-Geyda K., Maksimova Y., Gallagher P.G. (2013). Enhancers and Super Enhancers Are Associated with Genes That Control Phenotypic Traits In Primary Human Erythroid Cells. Blood.

[21] Majumder P., Lee J.T., Rahmberg A.R., Kumar G., Mi T., Scharer C.D., Boss J.M. (2020). A Super Enhancer Controls Expression and Chromatin Architecture within the MHC Class II Locus. J. Exp. Med..

[22] Quang D.X., Erdos M.R., Parker S.C.J., Collins F.S. (2015). Motif Signatures in Stretch Enhancers Are Enriched for Disease-Associated Genetic Variants. Epigenetics Chromatin.

[23] Vahedi G., Kanno Y., Furumoto Y., Jiang K., Parker S.C.J., Erdos M.R., Davis S.R., Roychoudhuri R., Restifo N.P., Gadina M. (2015). Super-Enhancers Delineate Disease-Associated Regulatory Nodes in T Cells. Nature.

[24] Khan A., Mathelier A., Zhang X. (2018). Super-Enhancers Are Transcriptionally More Active and Cell Type-Specific than Stretch Enhancers. Epigenetics.

[25] Dukler N., Gulko B., Huang Y.-F., Siepel A. (2017). Is a Super-Enhancer Greater than the Sum of Its Parts?. Nat. Genet..

[26] Hnisz D., Schuijers J., Lin C.Y., Weintraub A.S., Abraham B.J., Lee T.I., Bradner J.E., Young R.A. (2015). Convergence of Developmental and Oncogenic Signaling Pathways at Transcriptional Super-Enhancers. Mol. Cell.

[27] Khan A., Zhang X. (2016). DbSUPER: A Database of Super-Enhancers in Mouse and Human Genome. Nucleic Acids Res..

[28] Chen C., Zhou D., Gu Y., Wang C., Zhang M., Lin X., Xing J., Wang H., Zhang Y. (2019). SEA Version 3.0: A Comprehensive Extension and Update of the Super-Enhancer Archive. Nucleic Acids Res..

[29] Jiang Y., Qian F., Bai X., Liu Y., Wang Q., Ai B., Han X., Shi S., Zhang J., Li X. (2019). SEdb: A Comprehensive Human Super-Enhancer Database. Nucleic Acids Res..

[30] Wang X., Cairns M.J., Yan J. (2019). Super-Enhancers in Transcriptional Regulation and Genome Organization. Nucleic Acids Res..

[31] Ryu J., Kim H., Yang D., Lee A.J., Jung I. (2019). A New Class of Constitutively Active Super-Enhancers Is Associated with Fast Recovery of 3D Chromatin Loops. BMC Bioinform..

[32] Khan A., Zhang X. (2019). Integrative Modeling Reveals Key Chromatin and Sequence Signatures Predicting Super-Enhancers. Sci. Rep..

[33] Kursa M.B., Rudnicki W.R. (2010). Feature Selection with the Boruta Package. J. Stat. Soft..

[34] Bu H., Hao J., Gan Y., Zhou S., Guan J. (2019). DEEPSEN: A Convolutional Neural Network Based Method for Super-Enhancer Prediction. BMC Bioinform..

[35] Ji Q.-Y., Gong X.-J., Li H.-M., Du P.-F. (2021). DeepSE: Detecting Super-Enhancers among Typical Enhancers Using Only Sequence Feature Embeddings. Genomics.

[36] Gross D.S., Garrard W.T. (1988). Nuclease hypersensitive sites in chromatin. Annu. Rev. Biochem..

[37] Xi H., Shulha H.P., Lin J.M., Vales T.R., Fu Y., Bodine D.M., McKay R.D.G., Chenoweth J.G., Tesar P.J., Furey T.S. (2007). Identification and Characterization of Cell Type–Specific and Ubiquitous Chromatin Regulatory Structures in the Human Genome. PLoS Genet..

[38] Boyle A.P., Davis S., Shulha H.P., Meltzer P., Margulies E.H., Weng Z., Furey T.S., Crawford G.E. (2008). High-Resolution Mapping and Characterization of Open Chromatin across the Genome. Cell.

[39] Johnson D.S., Mortazavi A., Myers R.M., Wold B. (2007). Genome-Wide Mapping of in Vivo Protein-DNA Interactions. Science.

[40] Barski A., Cuddapah S., Cui K., Roh T.-Y., Schones D.E., Wang Z., Wei G., Chepelev I., Zhao K. (2007). High-Resolution Profiling of Histone Methylations in the Human Genome. Cell.

[41] Mikkelsen T.S., Ku M., Jaffe D.B., Issac B., Lieberman E., Giannoukos G., Alvarez P., Brockman W., Kim T.-K., Koche R.P. (2007). Genome-Wide Maps of Chromatin State in Pluripotent and Lineage-Committed Cells. Nature.

[42] Liu B., Liu X., Han L., Chen X., Wu X., Wu J., Yan D., Wang Y., Liu S., Shan L. (2022). BRD4-Directed Super-Enhancer Organization of Transcription Repression Programs Links to Chemotherapeutic Efficacy in Breast Cancer. Proc. Natl. Acad. Sci. USA.

[43] Visel A., Blow M.J., Li Z., Zhang T., Akiyama J.A., Holt A., Plajzer-Frick I., Shoukry M., Wright C., Chen F. (2009). ChIP-Seq Accurately Predicts Tissue-Specific Activity of Enhancers. Nature.

[44] Creyghton M.P., Cheng A.W., Welstead G.G., Kooistra T., Carey B.W., Steine E.J., Hanna J., Lodato M.A., Frampton G.M., Sharp P.A. (2010). Histone H3K27ac Separates Active from Poised Enhancers and Predicts Developmental State. Proc. Natl. Acad. Sci. USA.

[45] Buenrostro J.D., Giresi P.G., Zaba L.C., Chang H.Y., Greenleaf W.J. (2013). Transposition of Native Chromatin for Fast and Sensitive Epigenomic Profiling of Open Chromatin, DNA-Binding Proteins and Nucleosome Position. Nat. Methods.

[46] Honnell V., Norrie J.L., Patel A.G., Ramirez C., Zhang J., Lai Y.-H., Wan S., Dyer M.A. (2022). Identification of a Modular Super-Enhancer in Murine Retinal Development. Nat. Commun..

[47] Krishna V., Yin X., Song Q., Walsh A., Pocalyko D., Bachman K., Anderson I., Madakamutil L., Nagpal S. (2021). Integration of the Transcriptome and Genome-Wide Landscape of BRD2 and BRD4 Binding Motifs Identifies Key Superenhancer Genes and Reveals the Mechanism of Bet Inhibitor Action in Rheumatoid Arthritis Synovial Fibroblasts. J. Immunol..

[48] Yates K.B., Tonnerre P., Martin G.E., Gerdemann U., Al Abosy R., Comstock D.E., Weiss S.A., Wolski D., Tully D.C., Chung R.T. (2021). Epigenetic Scars of CD8^+^ T Cell Exhaustion Persist after Cure of Chronic Infection in Humans. Nat. Immunol..

[49] Giresi P.G., Kim J., McDaniell R.M., Iyer V.R., Lieb J.D. (2007). FAIRE (Formaldehyde-Assisted Isolation of Regulatory Elements) Isolates Active Regulatory Elements from Human Chromatin. Genome Res..

[50] Thakurela S., Sahu S.K., Garding A., Tiwari V.K. (2015). Dynamics and Function of Distal Regulatory Elements during Neurogenesis and Neuroplasticity. Genome Res..

[51] Core L.J., Waterfall J.J., Lis J.T. (2008). Nascent RNA Sequencing Reveals Widespread Pausing and Divergent Initiation at Human Promoters. Science.

[52] Lopes R., Reuven A., Gozde K. (2017). GRO-Seq, A Tool for Identification of Transcripts Regulating Gene Expression. Methods Mol. Biol..

[53] Meng F.-L., Du Z., Federation A., Hu J., Wang Q., Kieffer-Kwon K.-R., Meyers R.M., Amor C., Wasserman C.R., Neuberg D. (2014). Convergent Transcription at Intragenic Super-Enhancers Targets AID-Initiated Genomic Instability. Cell.

[54] Hah N., Benner C., Chong L.-W., Yu R.T., Downes M., Evans R.M. (2015). Inflammation-Sensitive Super Enhancers Form Domains of Coordinately Regulated Enhancer RNAs. Proc. Natl. Acad. Sci. USA.

[55] Lu Y., Shou J., Jia Z., Wu Y., Li J., Guo Y., Wu Q. (2019). Genetic Evidence for Asymmetric Blocking of Higher-Order Chromatin Structure by CTCF/Cohesin. Protein Cell.

[56] Li Y., Rivera C.M., Ishii H., Jin F., Selvaraj S., Lee A.Y., Dixon J.R., Ren B. (2014). CRISPR Reveals a Distal Super-Enhancer Required for Sox2 Expression in Mouse Embryonic Stem Cells. PLoS ONE.

[57] Zhang X., Choi P.S., Francis J.M., Imielinski M., Watanabe H., Cherniack A.D., Meyerson M. (2016). Identification of Focally Amplified Lineage-Specific Super-Enhancers in Human Epithelial Cancers. Nat. Genet..

[58] Cui S., Wu Q., Liu M., Su M., Liu S., Shao L., Han X., He H. (2021). EphA2 Super-Enhancer Promotes Tumor Progression by Recruiting FOSL2 and TCF7L2 to Activate the Target Gene EphA2. Cell Death Dis..

[59] Mushimiyimana I., Niskanen H., Beter M., Laakkonen J.P., Kaikkonen M.U., Ylä-Herttuala S., Laham-Karam N. (2021). Characterization of a Functional Endothelial Super-Enhancer That Regulates ADAMTS18 and Angiogenesis. Nucleic Acids Res..

[60] Guo Y., Xu Q., Canzio D., Shou J., Li J., Gorkin D.U., Jung I., Wu H., Zhai Y., Tang Y. (2015). CRISPR Inversion of CTCF Sites Alters Genome Topology and Enhancer/Promoter Function. Cell.

[61] Toyoda S., Kawaguchi M., Kobayashi T., Tarusawa E., Toyama T., Okano M., Oda M., Nakauchi H., Yoshimura Y., Sanbo M. (2014). Developmental Epigenetic Modification Regulates Stochastic Expression of Clustered Protocadherin Genes, Generating Single Neuron Diversity. Neuron.

[62] Monahan K., Horta A., Lomvardas S. (2019). LHX2- and LDB1-Mediated Trans Interactions Regulate Olfactory Receptor Choice. Nature.

[63] Mach P., Kos P.I., Zhan Y., Cramard J., Gaudin S., Tünnermann J., Marchi E., Eglinger J., Zuin J., Kryzhanovska M. (2022). Cohesin and CTCF Control the Dynamics of Chromosome Folding. Nat. Genet..

[64] Canzio D., Nwakeze C.L., Horta A., Rajkumar S.M., Coffey E.L., Duffy E.E., Duffié R., Monahan K., O’Keeffe S., Simon M.D. (2019). Antisense LncRNA Transcription Mediates DNA Demethylation to Drive Stochastic Protocadherin α Promoter Choice. Cell.

[65] Damaschke N.A., Gawdzik J., Avilla M., Yang B., Svaren J., Roopra A., Luo J.-H., Yu Y.P., Keles S., Jarrard D.F. (2020). CTCF Loss Mediates Unique DNA Hypermethylation Landscapes in Human Cancers. Clin. Epigenet..

[66] Ing-Simmons E., Seitan V.C., Faure A.J., Flicek P., Carroll T., Dekker J., Fisher A.G., Lenhard B., Merkenschlager M. (2015). Spatial Enhancer Clustering and Regulation of Enhancer-Proximal Genes by Cohesin. Genome Res..

[67] Willi M., Yoo K.H., Reinisch F., Kuhns T.M., Lee H.K., Wang C., Hennighausen L. (2017). Facultative CTCF Sites Moderate Mammary Super-Enhancer Activity and Regulate Juxtaposed Gene in Non-Mammary Cells. Nat. Commun.

[68] Vos E.S.M., Valdes-Quezada C., Huang Y., Allahyar A., Verstegen M.J.A.M., Felder A.-K., van der Vegt F., Uijttewaal E.C.H., Krijger P.H.L., de Laat W. (2021). Interplay between CTCF Boundaries and a Super Enhancer Controls Cohesin Extrusion Trajectories and Gene Expression. Mol. Cell.

[69] Jia Z., Li J., Ge X., Wu Y., Guo Y., Wu Q. (2020). Tandem CTCF Sites Function as Insulators to Balance Spatial Chromatin Contacts and Topological Enhancer-Promoter Selection. Genome Biol..

[70] Cho W.-K., Spille J.-H., Hecht M., Lee C., Li C., Grube V., Cisse I.I. (2018). Mediator and RNA Polymerase II Clusters Associate in Transcription-Dependent Condensates. Science.

[71] Sabari B.R., Dall’Agnese A., Boija A., Klein I.A., Coffey E.L., Shrinivas K., Abraham B.J., Hannett N.M., Zamudio A.V., Manteiga J.C. (2018). Coactivator Condensation at Super-Enhancers Links Phase Separation and Gene Control. Science.

[72] Lee R., Kang M.-K., Kim Y.-J., Yang B., Shim H., Kim S., Kim K., Yang C.M., Min B., Jung W.-J. (2022). CTCF-Mediated Chromatin Looping Provides a Topological Framework for the Formation of Phase-Separated Transcriptional Condensates. Nucleic Acids Res..

[73] Khoury A., Achinger-Kawecka J., Bert S.A., Smith G.C., French H.J., Luu P.-L., Peters T.J., Du Q., Parry A.J., Valdes-Mora F. (2020). Constitutively Bound CTCF Sites Maintain 3D Chromatin Architecture and Long-Range Epigenetically Regulated Domains. Nat. Commun..

[74] Bansal K., Michelson D.A., Ramirez R.N., Viny A.D., Levine R.L., Benoist C., Mathis D. (2021). Aire Regulates Chromatin Looping by Evicting CTCF from Domain Boundaries and Favoring Accumulation of Cohesin on Superenhancers. Proc. Natl. Acad. Sci. USA.

[75] Osterwalder M., Barozzi I., Tissières V., Fukuda-Yuzawa Y., Mannion B.J., Afzal S.Y., Lee E.A., Zhu Y., Plajzer-Frick I., Pickle C.S. (2018). Enhancer Redundancy Provides Phenotypic Robustness in Mammalian Development. Nature.

[76] Kai Y., Li B.E., Zhu M., Li G.Y., Chen F., Han Y., Cha H.J., Orkin S.H., Cai W., Huang J. (2021). Mapping the Evolving Landscape of Super-Enhancers during Cell Differentiation. Genome Biol..

[77] Liu X., Zhao B., Shaw T.I., Fridley B.L., Duckett D.R., Tan A.C., Teng M. (2022). Summarizing Internal Dynamics Boosts Differential Analysis and Functional Interpretation of Super Enhancers. Nucleic Acids Res..

[78] Vanhille L., Griffon A., Maqbool M.A., Zacarias-Cabeza J., Dao L.T.M., Fernandez N., Ballester B., Andrau J.C., Spicuglia S. (2015). High-Throughput and Quantitative Assessment of Enhancer Activity in Mammals by CapStarr-Seq. Nat. Commun..

[79] Bergman D.T., Jones T.R., Liu V., Ray J., Jagoda E., Siraj L., Kang H.Y., Nasser J., Kane M., Rios A. (2022). Compatibility Rules of Human Enhancer and Promoter Sequences. Nature.

[80] Kassouf M.T., Francis H.S., Gosden M., Suciu M.C., Downes D.J., Harrold C., Larke M., Oudelaar M., Cornell L., Blayney J. (2022). Multipartite Super-Enhancers Function in an Orientation-Dependent Manner. bioRxiv.

[81] Blayney J., Francis H., Camellato B., Mitchell L., Stolper R., Boeke J., Higgs D., Kassouf M. (2022). Super-Enhancers Require a Combination of Classical Enhancers and Novel Facilitator Elements to Drive High Levels of Gene Expression. bioRxiv.

[82] Wong E.S., Zheng D., Tan S.Z., Bower N.I., Garside V., Vanwalleghem G., Gaiti F., Scott E., Hogan B.M., Kikuchi K. (2020). Deep Conservation of the Enhancer Regulatory Code in Animals. Science.

[83] Villar D., Berthelot C., Aldridge S., Rayner T.F., Lukk M., Pignatelli M., Park T.J., Deaville R., Erichsen J.T., Jasinska A.J. (2015). Enhancer Evolution across 20 Mammalian Species. Cell.

[84] Flores M.A., Ovcharenko I. (2018). Enhancer Reprogramming in Mammalian Genomes. BMC Bioinform..

[85] Zboril E., Yoo H., Chen L., Liu Z. (2021). Dynamic Interactions of Transcription Factors and Enhancer Reprogramming in Cancer Progression. Front. Oncol..

[86] Bi M., Zhang Z., Jiang Y.-Z., Xue P., Wang H., Lai Z., Fu X., De Angelis C., Gong Y., Gao Z. (2020). Enhancer Reprogramming Driven by High-Order Assemblies of Transcription Factors Promotes Phenotypic Plasticity and Breast Cancer Endocrine Resistance. Nat. Cell Biol..

[87] Kamm G.B., Pisciottano F., Kliger R., Franchini L.F. (2013). The Developmental Brain Gene NPAS3 Contains the Largest Number of Accelerated Regulatory Sequences in the Human Genome. Mol. Biol. Evol..

[88] May D., Blow M.J., Kaplan T., McCulley D.J., Jensen B.C., Akiyama J.A., Holt A., Plajzer-Frick I., Shoukry M., Wright C. (2012). Large-Scale Discovery of Enhancers from Human Heart Tissue. Nat. Genet..

[89] Prabhakar S., Visel A., Akiyama J.A., Shoukry M., Lewis K.D., Holt A., Plajzer-Frick I., Morrison H., FitzPatrick D.R., Afzal V. (2008). Human-Specific Gain of Function in a Developmental Enhancer. Science.

[90] Sumiyama K., Saitou N. (2011). Loss-of-Function Mutation in a Repressor Module of Human-Specifically Activated Enhancer HACNS1. Mol. Biol. Evol..

[91] Capra J.A., Erwin G.D., McKinsey G., Rubenstein J.L.R., Pollard K.S. (2013). Many Human Accelerated Regions Are Developmental Enhancers. Phil. Trans. R. Soc. B.

[92] See Y.X., Chen K., Fullwood M.J. (2022). MYC Overexpression Leads to Increased Chromatin Interactions at Super-Enhancers and MYC Binding Sites. Genome Res..

[93] Oudelaar A.M., Davies J.O.J., Hanssen L.L.P., Telenius J.M., Schwessinger R., Liu Y., Brown J.M., Downes D.J., Chiariello A.M., Bianco S. (2018). Single-Allele Chromatin Interactions Identify Regulatory Hubs in Dynamic Compartmentalized Domains. Nat. Genet..

[94] Allahyar A., Vermeulen C., Bouwman B.A.M., Krijger P.H.L., Verstegen M.J.A.M., Geeven G., van Kranenburg M., Pieterse M., Straver R., Haarhuis J.H.I. (2018). Enhancer Hubs and Loop Collisions Identified from Single-Allele Topologies. Nat. Genet..

[95] Zhang J., Zhou Y., Yue W., Zhu Z., Wu X., Yu S., Shen Q., Pan Q., Xu W., Zhang R. (2022). Deeply Conserved Super-Enhancers Maintain Stem Cell Pluripotency in Placental Mammals. bioRxiv.

[96] Peng Y., Kang H., Luo J., Zhang Y. (2021). A Comparative Analysis of Super-Enhancers and Broad H3K4me3 Domains in Pig, Human, and Mouse Tissues. Front. Genet..

[97] Luan Y., Zhang L., Hu M., Xu Y., Hou Y., Li X., Zhao S., Zhao Y., Li C. (2019). Identification and Conservation Analysis of Cis-Regulatory Elements in Pig Liver. Genes.

[98] Pérez-Rico Y.A., Boeva V., Mallory A.C., Bitetti A., Majello S., Barillot E., Shkumatava A. (2017). Comparative Analyses of Super-Enhancers Reveal Conserved Elements in Vertebrate Genomes. Genome Res..

[99] Zhao Y., Hou Y., Xu Y., Luan Y., Zhou H., Qi X., Hu M., Wang D., Wang Z., Fu Y. (2021). A Compendium and Comparative Epigenomics Analysis of Cis-Regulatory Elements in the Pig Genome. Nat. Commun..

[100] Man J.C.K., van Duijvenboden K., Krijger P.H.L., Hooijkaas I.B., van der Made I., de Gier-de Vries C., Wakker V., Creemers E.E., de Laat W., Boukens B.J. (2021). Genetic Dissection of a Super Enhancer Controlling the *Nppa-Nppb* Cluster in the Heart. Circ. Res..

[101] Shiau C.-K., Huang J.-H., Liu Y.-T., Tsai H.-K. (2021). Genome-Wide Identification of Associations between Enhancer and Alternative Splicing in Human and Mouse. BMC Genom..

[102] Hazan I., Monin J., Bouwman B.A.M., Crosetto N., Aqeilan R.I. (2019). Activation of Oncogenic Super-Enhancers Is Coupled with DNA Repair by RAD51. Cell Rep..

[103] Wu Y., Yang Y., Gu H., Tao B., Zhang E., Wei J., Wang Z., Liu A., Sun R., Chen M. (2020). Multi-omics Analysis Reveals the Functional Transcription and Potential Translation of Enhancers. Int. J. Cancer.

[104] Rennie S., Dalby M., Lloret-Llinares M., Bakoulis S., Dalager Vaagensø C., Heick Jensen T., Andersson R. (2018). Transcription Start Site Analysis Reveals Widespread Divergent Transcription in D. Melanogaster and Core Promoter-Encoded Enhancer Activities. Nucleic Acids Res..

[105] Kainth A.S., Chowdhary S., Pincus D., Gross D.S. (2021). Primordial Super-Enhancers: Heat Shock-Induced Chromatin Organization in Yeast. Trends Cell Biol..

[106] Chowdhary S., Kainth A.S., Paracha S., Gross D.S., Pincus D. (2022). Inducible Transcriptional Condensates Drive 3D Genome Reorganization in the Heat Shock Response. Mol. Cell.

[107] Benton M.L., Talipineni S.C., Kostka D., Capra J.A. (2019). Genome-Wide Enhancer Annotations Differ Significantly in Genomic Distribution, Evolution, and Function. BMC Genom..

[108] Chen L., Fish A.E., Capra J.A. (2018). Prediction of Gene Regulatory Enhancers across Species Reveals Evolutionarily Conserved Sequence Properties. PLoS Comput. Biol..

[109] Young R.A. (2011). Control of the Embryonic Stem Cell State. Cell.

[110] Ng H.-H., Surani M.A. (2011). The Transcriptional and Signalling Networks of Pluripotency. Nat. Cell Biol..

[111] Tsai P.-H., Chien Y., Wang M.-L., Hsu C.-H., Laurent B., Chou S.-J., Chang W.-C., Chien C.-S., Li H.-Y., Lee H.-C. (2019). Ash2l Interacts with Oct4-Stemness Circuitry to Promote Super-Enhancer-Driven Pluripotency Network. Nucleic Acids Res..

[112] Zhou H.Y., Katsman Y., Dhaliwal N.K., Davidson S., Macpherson N.N., Sakthidevi M., Collura F., Mitchell J.A. (2014). A *Sox2* Distal Enhancer Cluster Regulates Embryonic Stem Cell Differentiation Potential. Genes Dev..

[113] Yue F., Cheng Y., Breschi A., Vierstra J., Wu W., Ryba T., Sandstrom R., Ma Z., Davis C., Pope B.D. (2014). A Comparative Encyclopedia of DNA Elements in the Mouse Genome. Nature.

[114] Blinka S., Reimer M.H., Pulakanti K., Rao S. (2016). Super-Enhancers at the Nanog Locus Differentially Regulate Neighboring Pluripotency-Associated Genes. Cell Rep..

[115] Di Micco R., Fontanals-Cirera B., Low V., Ntziachristos P., Yuen S.K., Lovell C.D., Dolgalev I., Yonekubo Y., Zhang G., Rusinova E. (2014). Control of Embryonic Stem Cell Identity by BRD4-Dependent Transcriptional Elongation of Super-Enhancer-Associated Pluripotency Genes. Cell Rep..

[116] Bortvin A., Winston F. (1996). Evidence That Spt6p Controls Chromatin Structure by a Direct Interaction with Histones. Science.

[117] Wang A.H., Juan A.H., Ko K.D., Tsai P.-F., Zare H., Dell’Orso S., Sartorelli V. (2017). The Elongation Factor Spt6 Maintains ESC Pluripotency by Controlling Super-Enhancers and Counteracting Polycomb Proteins. Mol. Cell.

[118] Zhang J., Zhou Y., Yue W., Zhu Z., Wu X., Yu S., Shen Q., Pan Q., Xu W., Zhang R. (2022). Super-Enhancers Conserved within Placental Mammals Maintain Stem Cell Pluripotency. Proc. Natl. Acad. Sci. USA.

[119] Wang C., Tian W., Hu S.-Y., Di C.-X., He C.-Y., Cao Q.-L., Hao R.-H., Dong S.-S., Liu C.-C., Rong Y. (2022). Lineage-Selective Super Enhancers Mediate Core Regulatory Circuitry during Adipogenic and Osteogenic Differentiation of Human Mesenchymal Stem Cells. Cell Death Dis..

[120] Huang Z., Jia B., Wang Q., Wang N., Zhao J. (2021). The Potential Function of Super Enhancers in Human Bone Marrow Mesenchymal Stem Cells during Osteogenic Differentiation. BioMed Res. Int..

[121] Factor D.C., Corradin O., Zentner G.E., Saiakhova A., Song L., Chenoweth J.G., McKay R.D., Crawford G.E., Scacheri P.C., Tesar P.J. (2014). Epigenomic Comparison Reveals Activation of “Seed” Enhancers during Transition from Naive to Primed Pluripotency. Cell Stem Cell.

[122] Lee B.-K., Jang Y., Kim M., LeBlanc L., Rhee C., Lee J., Beck S., Shen W., Kim J. (2019). Super-Enhancer-Guided Mapping of Regulatory Networks Controlling Mouse Trophoblast Stem Cells. Nat. Commun..

[123] Klein R.H., Hu W., Kashgari G., Lin Z., Nguyen T., Doan M., Andersen B. (2017). Characterization of Enhancers and the Role of the Transcription Factor KLF7 in Regulating Corneal Epithelial Differentiation. J. Biol. Chem..

[124] Siersbæk R., Rabiee A., Nielsen R., Sidoli S., Traynor S., Loft A., Poulsen L.L.C., Rogowska-Wrzesinska A., Jensen O.N., Mandrup S. (2014). Transcription Factor Cooperativity in Early Adipogenic Hotspots and Super-Enhancers. Cell Rep..

[125] Brunmeir R., Wu J., Peng X., Kim S.-Y., Julien S.G., Zhang Q., Xie W., Xu F. (2016). Comparative Transcriptomic and Epigenomic Analyses Reveal New Regulators of Murine Brown Adipogenesis. PLoS Genet..

[126] Huang J., Liu X., Li D., Shao Z., Cao H., Zhang Y., Trompouki E., Bowman T.V., Zon L.I., Yuan G.-C. (2016). Dynamic Control of Enhancer Repertoires Drives Lineage and Stage-Specific Transcription during Hematopoiesis. Dev. Cell.

[127] Aranda-Orgilles B., Saldaña-Meyer R., Wang E., Trompouki E., Fassl A., Lau S., Mullenders J., Rocha P.P., Raviram R., Guillamot M. (2016). MED12 Regulates HSC-Specific Enhancers Independently of Mediator Kinase Activity to Control Hematopoiesis. Cell Stem Cell.

[128] Liu C.-F., Lefebvre V. (2015). The Transcription Factors SOX9 and SOX5/SOX6 Cooperate Genome-Wide through Super-Enhancers to Drive Chondrogenesis. Nucleic Acids Res..

[129] Ang Y.-S., Rivas R.N., Ribeiro A.J.S., Srivas R., Rivera J., Stone N.R., Pratt K., Mohamed T.M.A., Fu J.-D., Spencer C.I. (2016). Disease Model of GATA4 Mutation Reveals Transcription Factor Cooperativity in Human Cardiogenesis. Cell.

[130] Zhao Y., Zhou J., He L., Li Y., Yuan J., Sun K., Chen X., Bao X., Esteban M.A., Sun H. (2019). MyoD Induced Enhancer RNA Interacts with HnRNPL to Activate Target Gene Transcription during Myogenic Differentiation. Nat. Commun..

[131] Caputo V.S., Trasanidis N., Xiao X., Robinson M.E., Katsarou A., Ponnusamy K., Prinjha R.K., Smithers N., Chaidos A., Auner H.W. (2021). Brd2/4 and Myc Regulate Alternative Cell Lineage Programmes during Early Osteoclast Differentiation in Vitro. iScience.

[132] Horie M., Miyashita N., Mikami Y., Noguchi S., Yamauchi Y., Suzukawa M., Fukami T., Ohta K., Asano Y., Sato S. (2018). TBX4 Is Involved in the Super-Enhancer-Driven Transcriptional Programs Underlying Features Specific to Lung Fibroblasts. Am. J. Physiol.-Lung Cell. Mol. Physiol..

[133] Paraiso K.D., Blitz I.L., Coley M., Cheung J., Sudou N., Taira M., Cho K.W.Y. (2019). Endodermal Maternal Transcription Factors Establish Super-Enhancers during Zygotic Genome Activation. Cell Rep..

[134] Kitagawa Y., Ohkura N., Kidani Y., Vandenbon A., Hirota K., Kawakami R., Yasuda K., Motooka D., Nakamura S., Kondo M. (2017). Guidance of Regulatory T Cell Development by Satb1-Dependent Super-Enhancer Establishment. Nat. Immunol..

[135] Thomas G.D., Hanna R.N., Vasudevan N.T., Hamers A.A., Romanoski C.E., McArdle S., Ross K.D., Blatchley A., Yoakum D., Hamilton B.A. (2016). Deleting an Nr4a1 Super-Enhancer Subdomain Ablates Ly6C Low Monocytes While Preserving Macrophage Gene Function. Immunity.

[136] Shin H.Y., Willi M., Yoo K.H., Zeng X., Wang C., Metser G., Hennighausen L. (2016). Hierarchy within the Mammary STAT5-Driven Wap Super-Enhancer. Nat. Genet..

[137] Lee H.K., Willi M., Shin H.Y., Liu C., Hennighausen L. (2018). Progressing Super-Enhancer Landscape during Mammary Differentiation Controls Tissue-Specific Gene Regulation. Nucleic Acids Res..

[138] Martinez M.F., Medrano S., Brown E.A., Tufan T., Shang S., Bertoncello N., Guessoum O., Adli M., Belyea B.C., Sequeira-Lopez M.L.S. (2018). Super-Enhancers Maintain Renin-Expressing Cell Identity and Memory to Preserve Multi-System Homeostasis. J. Clin. Investig..

[139] Alvarez-Dominguez J.R., Knoll M., Gromatzky A.A., Lodish H.F. (2017). The Super-Enhancer-Derived AlncRNA-EC7/Bloodlinc Potentiates Red Blood Cell Development in Trans. Cell Rep..

[140] Dubois-Chevalier J., Dubois V., Staels B., Lefebvre P., Eeckhoute J. (2020). Perspectives on the Use of Super-Enhancers as a Defining Feature of Cell/Tissue-Identity Genes. Epigenomics.

[141] Nott A., Holtman I.R., Coufal N.G., Schlachetzki J.C.M., Yu M., Hu R., Han C.Z., Pena M., Xiao J., Wu Y. (2019). Brain Cell Type–Specific Enhancer–Promoter Interactome Maps and Disease—Risk Association. Science.

[142] Achour M., Le Gras S., Keime C., Parmentier F., Lejeune F.-X., Boutillier A.-L., Neri C., Davidson I., Merienne K. (2015). Neuronal Identity Genes Regulated by Super-Enhancers Are Preferentially down-Regulated in the Striatum of Huntington’s Disease Mice. Hum. Mol. Genet..

[143] Brown J.D., Lin C.Y., Duan Q., Griffin G., Federation A.J., Paranal R.M., Bair S., Newton G., Lichtman A.H., Kung A.L. (2014). NF-ΚB Directs Dynamic Super Enhancer Formation in Inflammation and Atherogenesis. Mol. Cell.

[144] Schmidt S.F., Larsen B.D., Loft A., Nielsen R., Madsen J.G.S., Mandrup S. (2015). Acute TNF-Induced Repression of Cell Identity Genes Is Mediated by NFκB-Directed Redistribution of Cofactors from Super-Enhancers. Genome Res..

[145] Higashijima Y., Matsui Y., Shimamura T., Nakaki R., Nagai N., Tsutsumi S., Abe Y., Link V.M., Osaka M., Yoshida M. (2020). Coordinated Demethylation of H3K9 and H3K27 Is Required for Rapid Inflammatory Responses of Endothelial Cells. EMBO J..

[146] Xiao X., Fan Y., Li J., Zhang X., Lou X., Dou Y., Shi X., Lan P., Xiao Y., Minze L. (2018). Guidance of Super-Enhancers in Regulation of IL-9 Induction and Airway Inflammation. J. Exp. Med..

[147] Peeters J.G.C., Vervoort S.J., Tan S.C., Mijnheer G., de Roock S., Vastert S.J., Nieuwenhuis E.E.S., van Wijk F., Prakken B.J., Creyghton M.P. (2015). Inhibition of Super-Enhancer Activity in Autoinflammatory Site-Derived T Cells Reduces Disease-Associated Gene Expression. Cell Rep..

[148] Rakyan V.K., Down T.A., Balding D.J., Beck S. (2011). Epigenome-Wide Association Studies for Common Human Diseases. Nat. Rev. Genet..

[149] Lacey M., Baribault C., Ehrlich K.C., Ehrlich M. (2019). Atherosclerosis-Associated Differentially Methylated Regions Can Reflect the Disease Phenotype and Are Often at Enhancers. Atherosclerosis.

[150] Sun W., Yao S., Tang J., Liu S., Chen J., Deng D., Zeng C. (2018). Integrative Analysis of Super Enhancer SNPs for Type 2 Diabetes. PLoS ONE.

[151] Kubota S., Tokunaga K., Umezu T., Yokomizo-Nakano T., Sun Y., Oshima M., Tan K.T., Yang H., Kanai A., Iwanaga E. (2019). Lineage-Specific RUNX2 Super-Enhancer Activates MYC and Promotes the Development of Blastic Plasmacytoid Dendritic Cell Neoplasm. Nat. Commun..

[152] Gröschel S., Sanders M.A., Hoogenboezem R., de Wit E., Bouwman B.A.M., Erpelinck C., van der Velden V.H.J., Havermans M., Avellino R., van Lom K. (2014). A Single Oncogenic Enhancer Rearrangement Causes Concomitant EVI1 and GATA2 Deregulation in Leukemia. Cell.

[153] Affer M., Chesi M., Chen W.D., Keats J.J., Demchenko Y.N., Tamizhmani K., Garbitt V.M., Riggs D.L., Brents L.A., Roschke A.V. (2014). Promiscuous MYC Locus Rearrangements Hijack Enhancers but Mostly Super-Enhancers to Dysregulate MYC Expression in Multiple Myeloma. Leukemia.

[154] Drier Y., Cotton M.J., Williamson K.E., Gillespie S.M., Ryan R.J.H., Kluk M.J., Carey C.D., Rodig S.J., Sholl L.M., Afrogheh A.H. (2016). An Oncogenic MYB Feedback Loop Drives Alternate Cell Fates in Adenoid Cystic Carcinoma. Nat. Genet..

[155] Jiang Y., Jiang Y.-Y., Lin D.-C. (2021). Super-Enhancer-Mediated Core Regulatory Circuitry in Human Cancer. Comput. Struct. Biotechnol. J..

[156] (2017). The Cancer Genome Atlas Research Network Integrated Genomic Characterization of Oesophageal Carcinoma. Nature.

[157] Hao J.-J., Lin D.-C., Dinh H.Q., Mayakonda A., Jiang Y.-Y., Chang C., Jiang Y., Lu C.-C., Shi Z.-Z., Xu X. (2016). Spatial Intratumoral Heterogeneity and Temporal Clonal Evolution in Esophageal Squamous Cell Carcinoma. Nat. Genet..

[158] Lin D.-C., Wang M.-R., Koeffler H.P. (2018). Genomic and Epigenomic Aberrations in Esophageal Squamous Cell Carcinoma and Implications for Patients. Gastroenterology.

[159] Cao W., Wu W., Yan M., Tian F., Ma C., Zhang Q., Li X., Han P., Liu Z., Gu J. (2015). Multiple Region Whole-Exome Sequencing Reveals Dramatically Evolving Intratumor Genomic Heterogeneity in Esophageal Squamous Cell Carcinoma. Oncogenesis.

[160] Chapuy B., McKeown M.R., Lin C.Y., Monti S., Roemer M.G.M., Qi J., Rahl P.B., Sun H.H., Yeda K.T., Doench J.G. (2013). Discovery and Characterization of Super-Enhancer-Associated Dependencies in Diffuse Large B Cell Lymphoma. Cancer Cell.

[161] Betancur P.A., Abraham B.J., Yiu Y.Y., Willingham S.B., Khameneh F., Zarnegar M., Kuo A.H., McKenna K., Kojima Y., Leeper N.J. (2017). A CD47-Associated Super-Enhancer Links pro-Inflammatory Signalling to CD47 Upregulation in Breast Cancer. Nat. Commun..

[162] Chen H., Liang H. (2020). A High-Resolution Map of Human Enhancer RNA Loci Characterizes Super-Enhancer Activities in Cancer. Cancer Cell.

[163] Kelly M.R., Wisniewska K., Regner M.J., Lewis M.W., Perreault A.A., Davis E.S., Phanstiel D.H., Parker J.S., Franco H.L. (2022). A Multi-Omic Dissection of Super-Enhancer Driven Oncogenic Gene Expression Programs in Ovarian Cancer. Nat. Commun..

[164] Yokoyama Y., Zhu H., Lee J.H., Kossenkov A.V., Wu S.Y., Wickramasinghe J.M., Yin X., Palozola K.C., Gardini A., Showe L.C. (2016). BET Inhibitors Suppress ALDH Activity by Targeting *ALDH1A1* Super-Enhancer in Ovarian Cancer. Cancer Res..

[165] Pfeffer S.R., Yang C.H., Pfeffer L.M. (2015). The Role of MiR-21 in Cancer: The Role of MiR-21 In Cancer. Drug Dev. Res..

[166] Kumarswamy R., Volkmann I., Thum T. (2011). Regulation and Function of MiRNA-21 in Health and Disease. RNA Biol..

[167] Fujita S., Ito T., Mizutani T., Minoguchi S., Yamamichi N., Sakurai K., Iba H. (2008). MiR-21 Gene Expression Triggered by AP-1 Is Sustained through a Double-Negative Feedback Mechanism. J. Mol. Biol..

[168] Ribas J., Lupold S.E. (2010). The Transcriptional Regulation of MiR-21, Its Multiple Transcripts and Their Implication in Prostate Cancer. Cell Cycle.

[169] Wan Y., Hoyle R.G., Xie N., Wang W., Cai H., Zhang M., Ma Z., Xiong G., Xu X., Huang Z. (2021). A Super-Enhancer Driven by FOSL1 Controls MiR-21-5p Expression in Head and Neck Squamous Cell Carcinoma. Front. Oncol..

[170] Jang M.K., Mochizuki K., Zhou M., Jeong H.-S., Brady J.N., Ozato K. (2005). The Bromodomain Protein Brd4 Is a Positive Regulatory Component of P-TEFb and Stimulates RNA Polymerase II-Dependent Transcription. Mol. Cell.

[171] Fisher R.P., Morgan D.O. (1994). A Novel Cyclin Associates with M015/CDK7 to Form the CDK-Activating Kinase. Cell.

[172] Dahmus M.E. (1996). Reversible Phosphorylation of the C-Terminal Domain of RNA Polymerase II. J. Biol. Chem..

[173] Filippakopoulos P., Picaud S., Mangos M., Keates T., Lambert J.-P., Barsyte-Lovejoy D., Felletar I., Volkmer R., Müller S., Pawson T. (2012). Histone Recognition and Large-Scale Structural Analysis of the Human Bromodomain Family. Cell.

[174] Filippakopoulos P., Qi J., Picaud S., Shen Y., Smith W.B., Fedorov O., Morse E.M., Keates T., Hickman T.T., Felletar I. (2010). Selective Inhibition of BET Bromodomains. Nature.

[175] Tolani B., Gopalakrishnan R., Punj V., Matta H., Chaudhary P.M. (2014). Targeting Myc in KSHV-Associated Primary Effusion Lymphoma with BET Bromodomain Inhibitors. Oncogene.

[176] Sengupta D., Kannan A., Kern M., Moreno M.A., Vural E., Stack B., Suen J.Y., Tackett A.J., Gao L. (2015). Disruption of BRD4 at H3K27Ac-Enriched Enhancer Region Correlates with Decreased c-Myc Expression in Merkel Cell Carcinoma. Epigenetics.

[177] Tögel L., Nightingale R., Chueh A.C., Jayachandran A., Tran H., Phesse T., Wu R., Sieber O.M., Arango D., Dhillon A.S. (2016). Dual Targeting of Bromodomain and Extraterminal Domain Proteins, and WNT or MAPK Signaling, Inhibits c-MYC Expression and Proliferation of Colorectal Cancer Cells. Mol. Cancer Ther..

[178] Chen D., Zhao Z., Huang Z., Chen D.-C., Zhu X.-X., Wang Y.-Z., Yan Y.-W., Tang S., Madhavan S., Ni W. (2018). Super Enhancer Inhibitors Suppress MYC Driven Transcriptional Amplification and Tumor Progression in Osteosarcoma. Bone Res..

[179] Murakami S., Li R., Nagari A., Chae M., Camacho C.V., Kraus W.L. (2019). Distinct Roles for BET Family Members in Estrogen Receptor α Enhancer Function and Gene Regulation in Breast Cancer Cells. Mol. Cancer Res..

[180] Sin-Chan P., Mumal I., Suwal T., Ho B., Fan X., Singh I., Du Y., Lu M., Patel N., Torchia J. (2019). A C19MC-LIN28A-MYCN Oncogenic Circuit Driven by Hijacked Super-Enhancers Is a Distinct Therapeutic Vulnerability in ETMRs: A Lethal Brain Tumor. Cancer Cell.

[181] Nagasawa M., Tomimatsu K., Terada K., Kondo K., Miyazaki K., Miyazaki M., Motooka D., Okuzaki D., Yoshida T., Kageyama S. (2020). Long Non-Coding RNA MANCR Is a Target of BET Bromodomain Protein BRD4 and Plays a Critical Role in Cellular Migration and Invasion Abilities of Prostate Cancer. Biochem. Biophys. Res. Commun..

[182] Nicodeme E., Jeffrey K.L., Schaefer U., Beinke S., Dewell S., Chung C., Chandwani R., Marazzi I., Wilson P., Coste H. (2010). Suppression of Inflammation by a Synthetic Histone Mimic. Nature.

[183] Shu S., Lin C.Y., He H.H., Witwicki R.M., Tabassum D.P., Roberts J.M., Janiszewska M., Jin Huh S., Liang Y., Ryan J. (2016). Response and Resistance to BET Bromodomain Inhibitors in Triple-Negative Breast Cancer. Nature.

[184] Gibbons H.R., Mi D.J., Farley V.M., Esmond T., Kaood M.B., Aune T.M. (2019). Bromodomain Inhibitor JQ1 Reversibly Blocks IFN-γ Production. Sci. Rep..

[185] Kwiatkowski N., Zhang T., Rahl P.B., Abraham B.J., Reddy J., Ficarro S.B., Dastur A., Amzallag A., Ramaswamy S., Tesar B. (2014). Targeting Transcription Regulation in Cancer with a Covalent CDK7 Inhibitor. Nature.

[186] Christensen C.L., Kwiatkowski N., Abraham B.J., Carretero J., Al-Shahrour F., Zhang T., Chipumuro E., Herter-Sprie G.S., Akbay E.A., Altabef A. (2014). Targeting Transcriptional Addictions in Small Cell Lung Cancer with a Covalent CDK7 Inhibitor. Cancer Cell.

[187] Eliades P., Abraham B.J., Ji Z., Miller D.M., Christensen C.L., Kwiatkowski N., Kumar R., Njauw C.N., Taylor M., Miao B. (2018). High MITF Expression Is Associated with Super-Enhancers and Suppressed by CDK7 Inhibition in Melanoma. J. Investig. Dermatol..

[188] Chipumuro E., Marco E., Christensen C.L., Kwiatkowski N., Zhang T., Hatheway C.M., Abraham B.J., Sharma B., Yeung C., Altabef A. (2014). CDK7 Inhibition Suppresses Super-Enhancer-Linked Oncogenic Transcription in MYCN-Driven Cancer. Cell.

[189] Wang Y., Zhang T., Kwiatkowski N., Abraham B.J., Lee T.I., Xie S., Yuzugullu H., Von T., Li H., Lin Z. (2015). CDK7-Dependent Transcriptional Addiction in Triple-Negative Breast Cancer. Cell.

[190] Zhao X., Ren Y., Lawlor M., Shah B.D., Park P.M.C., Lwin T., Wang X., Liu K., Wang M., Gao J. (2019). BCL2 Amplicon Loss and Transcriptional Remodeling Drives ABT-199 Resistance in B Cell Lymphoma Models. Cancer Cell.

[191] Sanchez G.J., Richmond P.A., Bunker E.N., Karman S.S., Azofeifa J., Garnett A.T., Xu Q., Wheeler G.E., Toomey C.M., Zhang Q. (2018). Genome-Wide Dose-Dependent Inhibition of Histone Deacetylases Studies Reveal Their Roles in Enhancer Remodeling and Suppression of Oncogenic Super-Enhancers. Nucleic Acids Res..

[192] Gryder B.E., Pomella S., Sayers C., Wu X.S., Song Y., Chiarella A.M., Bagchi S., Chou H.-C., Sinniah R.S., Walton A. (2019). Histone Hyperacetylation Disrupts Core Gene Regulatory Architecture in Rhabdomyosarcoma. Nat. Genet..

[193] Caslini C., Hong S., Ban Y.J., Chen X.S., Ince T.A. (2019). HDAC7 Regulates Histone 3 Lysine 27 Acetylation and Transcriptional Activity at Super-Enhancer-Associated Genes in Breast Cancer Stem Cells. Oncogene.

[194] Gryder B.E., Wu L., Woldemichael G.M., Pomella S., Quinn T.R., Park P.M.C., Cleveland A., Stanton B.Z., Song Y., Rota R. (2019). Chemical Genomics Reveals Histone Deacetylases Are Required for Core Regulatory Transcription. Nat. Commun..

[195] Shi K., Yin X., Cai M.-C., Yan Y., Jia C., Ma P., Zhang S., Zhang Z., Gu Z., Zhang M. (2019). PAX8 Regulon in Human Ovarian Cancer Links Lineage Dependency with Epigenetic Vulnerability to HDAC Inhibitors. eLife.

[196] Nguyen T.T.T., Zhang Y., Shang E., Shu C., Torrini C., Zhao J., Bianchetti E., Mela A., Humala N., Mahajan A. (2020). HDAC Inhibitors Elicit Metabolic Reprogramming by Targeting Super-Enhancers in Glioblastoma Models. J. Clin. Investig..

[197] Sugino N., Kawahara M., Tatsumi G., Kanai A., Matsui H., Yamamoto R., Nagai Y., Fujii S., Shimazu Y., Hishizawa M. (2017). A Novel LSD1 Inhibitor NCD38 Ameliorates MDS-Related Leukemia with Complex Karyotype by Attenuating Leukemia Programs via Activating Super-Enhancers. Leukemia.

[198] Tatsumi G., Kawahara M., Yamamoto R., Hishizawa M., Kito K., Suzuki T., Takaori-Kondo A., Andoh A. (2020). LSD1-Mediated Repression of GFI1 Super-Enhancer Plays an Essential Role in Erythroleukemia. Leukemia.

[199] Zhang J., Ying Y., Li M., Wang M., Huang X., Jia M., Zeng J., Ma C., Zhang Y., Li C. (2020). Targeted Inhibition of KDM6 Histone Demethylases Eradicates Tumor-Initiating Cells via Enhancer Reprogramming in Colorectal Cancer. Theranostics.

[200] Meloche S., Pouysségur J. (2007). The ERK1/2 Mitogen-Activated Protein Kinase Pathway as a Master Regulator of the G1- to S-Phase Transition. Oncogene.

[201] Hilger R.A., Scheulen M.E., Strumberg D. (2002). The Ras-Raf-MEK-ERK Pathway in the Treatment of Cancer. Oncol. Res. Treat..

[202] Yohe M.E., Gryder B.E., Shern J.F., Song Y.K., Chou H.-C., Sindiri S., Mendoza A., Patidar R., Zhang X., Guha R. (2018). MEK Inhibition Induces MYOG and Remodels Super-Enhancers in RAS-Driven Rhabdomyosarcoma. Sci. Transl. Med..

[203] Pomella S., Sreenivas P., Gryder B.E., Wang L., Milewski D., Cassandri M., Baxi K., Hensch N.R., Carcarino E., Song Y. (2021). Interaction between SNAI2 and MYOD Enhances Oncogenesis and Suppresses Differentiation in Fusion Negative Rhabdomyosarcoma. Nat. Commun..

[204] Mack S.C., Singh I., Wang X., Hirsch R., Wu Q., Villagomez R., Bernatchez J.A., Zhu Z., Gimple R.C., Kim L.J.Y. (2019). Chromatin Landscapes Reveal Developmentally Encoded Transcriptional States That Define Human Glioblastoma. J. Exp. Med..

[205] Furqan M., Mukhi N., Lee B., Liu D. (2013). Dysregulation of JAK-STAT Pathway in Hematological Malignancies and JAK Inhibitors for Clinical Application. Biomark. Res..

[206] Pelish H.E., Liau B.B., Nitulescu I.I., Tangpeerachaikul A., Poss Z.C., Da Silva D.H., Caruso B.T., Arefolov A., Fadeyi O., Christie A.L. (2015). Mediator Kinase Inhibition Further Activates Super-Enhancer-Associated Genes in AML. Nature.

[207] Call S.G., Duren R.P., Panigrahi A.K., Nguyen L., Freire P.R., Grimm S.L., Coarfa C., Conneely O.M. (2020). Targeting Oncogenic Super Enhancers in MYC-Dependent AML Using a Small Molecule Activator of NR4A Nuclear Receptors. Sci. Rep..

[208] Bolin S., Borgenvik A., Persson C.U., Sundström A., Qi J., Bradner J.E., Weiss W.A., Cho Y.-J., Weishaupt H., Swartling F.J. (2018). Combined BET Bromodomain and CDK2 Inhibition in MYC-Driven Medulloblastoma. Oncogene.

[209] Wiese M., Hamdan F.H., Kubiak K., Diederichs C., Gielen G.H., Nussbaumer G., Carcaboso A.M., Hulleman E., Johnsen S.A., Kramm C.M. (2020). Combined Treatment with CBP and BET Inhibitors Reverses Inadvertent Activation of Detrimental Super Enhancer Programs in DIPG Cells. Cell Death Dis..

[210] Chen Z., Cao Z., Wang Y., Zhang X., Xu L., Wang Y., Chen Y., Yang C., Ding J., Meng L. (2022). Repressing MYC by Targeting BET Synergizes with Selective Inhibition of PI3Kα against B Cell Lymphoma. Cancer Lett..

[211] Tee A.E., Ciampa O.C., Wong M., Fletcher J.I., Kamili A., Chen J., Ho N., Sun Y., Carter D.R., Cheung B.B. (2020). Combination Therapy with the CDK7 Inhibitor and the Tyrosine Kinase Inhibitor Exerts Synergistic Anticancer Effects against *MYCN* -amplified Neuroblastoma. Int. J. Cancer.

[212] Shang E., Nguyen T.T.T., Shu C., Westhoff M.-A., Karpel-Massler G., Siegelin M.D. (2020). Epigenetic Targeting of Mcl-1 Is Synthetically Lethal with Bcl-XL/Bcl-2 Inhibition in Model Systems of Glioblastoma. Cancers.

[213] Nagaraja S., Vitanza N.A., Woo P.J., Taylor K.R., Liu F., Zhang L., Li M., Meng W., Ponnuswami A., Sun W. (2017). Transcriptional Dependencies in Diffuse Intrinsic Pontine Glioma. Cancer Cell.

[214] Wong M., Sun Y., Xi Z., Milazzo G., Poulos R.C., Bartenhagen C., Bell J.L., Mayoh C., Ho N., Tee A.E. (2019). JMJD6 Is a Tumorigenic Factor and Therapeutic Target in Neuroblastoma. Nat. Commun..

[215] Shen Z., Bao W., Huang D.-S. (2018). Recurrent Neural Network for Predicting Transcription Factor Binding Sites. Sci. Rep..

[216] Ji Y., Zhou Z., Liu H., Davuluri R.V. (2021). DNABERT: Pre-Trained Bidirectional Encoder Representations from Transformers Model for DNA-Language in Genome. Bioinformatics.

[217] Spirin P.V., Lebedev T.D., Orlova N.N., Gornostaeva A.S., Prokofjeva M.M., Nikitenko N.A., Dmitriev S.E., Buzdin A.A., Borisov N.M., Aliper A.M. (2014). Silencing AML1-ETO Gene Expression Leads to Simultaneous Activation of Both pro-Apoptotic and Proliferation Signaling. Leukemia.

[218] Lebedev T.D., Spirin P.V., Orlova N.N., Prokofjeva M.M., Prassolov V.S. (2015). Comparative Analysis of Gene Expression: Targeted Antitumor Therapy in Neuroblastoma Cell Lines. Mol. Biol..

[219] Mitkevich V.A., Orlova N.N., Petrushanko I.Y., Simonenko O.V., Spirin P.V., Prokof’eva M.M., Gornostaeva A.S., Stocking C., Makarov A.A., Prassolov V.S. (2013). Expression of the FLT3-ITD Oncogene Sensitizes Murine Progenitor B-Cell Line BAF3 to Cytotoxic Action of Binase. Mol. Biol..

[220] Orlova N.N., Lebedev T.D., Spirin P.V., Prassolov V.S. (2016). Key Molecular Mechanisms Associated with Cell Malignant Transformation in Acute Myeloid Leukemia. Mol. Biol..

[221] Ivanov A.E., Pushkarev A.V., Orlov A.V., Nikitin M.P., Nikitin P.I. (2019). Interferometric Detection of Chloramphenicol via Its Immunochemical Recognition at Polymer-Coated Nano-Corrugated Surfaces. Sens. Actuators B Chem..

[222] Novichikhin D.O., Orlov A.V., Antopolsky M.L., Znoyko S.L., Nikitin P.I. (2022). Specific and Sensitive Determination of Folic Acid by Label-Free Chemosensors with Microscope Glass Slips as Single-Use Consumables. Chemosensors.

[223] Orlov A.V., Novichikhin D.O., Pushkarev A.V., Malkerov Y.A., Znoiko S.L., Guteneva N.V., Orlova N.N., Gorshkov B.G., Nikitin P.I. (2022). Registering the Kinetics of Intermolecular Interactions by Low-Coherence Interferometry for the Development of Biomarker Immunoassays for Cardiovascular Diseases. Dokl. Phys..

[224] Bragina V.A., Orlov A.V., Znoyko S.L., Pushkarev A.V., Novichikhin D.O., Guteneva N.V., Nikitin M.P., Gorshkov B.G., Nikitin P.I. (2021). Nanobiosensing Based on Optically Selected Antibodies and Superparamagnetic Labels for Rapid and Highly Sensitive Quantification of Polyvalent Hepatitis B Surface Antigen. Anal. Methods.

[225] Pushkarev A.V., Orlov A.V., Znoyko S.L., Bragina V.A., Nikitin P.I. (2021). Rapid and Easy-to-Use Method for Accurate Characterization of Target Binding and Kinetics of Magnetic Particle Bioconjugates for Biosensing. Sensors.

[226] Orlov A.V., Malkerov J.A., Novichikhin D.O., Znoyko S.L., Nikitin P.I. (2022). Express High-Sensitive Detection of Ochratoxin A in Food by a Lateral Flow Immunoassay Based on Magnetic Biolabels. Food Chem..

[227] Orlov A.V., Malkerov J.A., Novichikhin D.O., Znoyko S.L., Nikitin P.I. (2022). Multiplex Label-Free Kinetic Characterization of Antibodies for Rapid Sensitive Cardiac Troponin I Detection Based on Functionalized Magnetic Nanotags. Int. J. Mol. Sci..

[228] Burenin A.G., Nikitin M.P., Orlov A.V., Ksenevich T.I., Nikitin P.I. (2013). Detection of Pyrethroids by Spectral Correlation Interferometry. Appl. Biochem. Microbiol..

[229] Nekrasov N., Kudriavtseva A., Orlov A.V., Gadjanski I., Nikitin P.I., Bobrinetskiy I., Knežević N.Ž. (2022). One-Step Photochemical Immobilization of Aptamer on Graphene for Label-Free Detection of NT-ProBNP. Biosensors.

[230] Nekrasov N., Yakunina N., Pushkarev A.V., Orlov A.V., Gadjanski I., Pesquera A., Centeno A., Zurutuza A., Nikitin P.I., Bobrinetskiy I. (2021). Spectral-Phase Interferometry Detection of Ochratoxin a via Aptamer-Functionalized Graphene Coated Glass. Nanomaterials.

[231] Orlova N.N., Bogatova O.V., Orlov A.V. (2020). High-Performance Method for Identification of Super Enhancers from ChIP-Seq Data with Configurable Cloud Virtual Machines. MethodsX.

[232] Orlova N.N., Nikitina A.S., Orlov A.V. (2019). Identification and Analysis of Super-Enhancers as Novel Biomarkers and Potential Therapeutic Targets for Age-Associated Diseases. FEBS Open Bio..

